# Composition and Genetic Diversity of Mosquitoes (Diptera: Culicidae) on Islands and Mainland Shores of Kenya’s Lakes Victoria and Baringo

**DOI:** 10.1093/jme/tjw102

**Published:** 2016-07-11

**Authors:** Yvonne Ukamaka Ajamma, Jandouwe Villinger, David Omondi, Daisy Salifu, Thomas Ogao Onchuru, Laban Njoroge, Anne W. T. Muigai, Daniel K. Masiga

**Affiliations:** ^1^Martin Lüscher Emerging Infectious Diseases (ML-EID) Laboratory, International Centre of Insect Physiology and Ecology, Kasarani, P. O. Box 30772-00100, Nairobi, Kenya (yvonneuo@yahoo.com; jandouwe@icipe.org; domondi@icipe.org; dsalifu@icipe.org; ogathomas@gmail.com; dmasiga@icipe.org); ^2^Department of Botany (Genetics), Jomo Kenyatta University of Agriculture and Technology, Juja, P. O. Box 62000-00200, Nairobi, Kenya (awmuigai@yahoo.co.uk); ^4^Biochemistry and Molecular Biology Department, Egerton University, P. O. Box 536-20115, Egerton, Kenya; ^5^Molecular Biology and Virology Laboratory, Department of Medical Biosciences, University of Western Cape, Private Bag X17, Bellville 7535, South Africa; ^6^Insect Symbiosis Research Group, Max Planck Institute for Chemical Ecology (MPI-CE), Hans-Knoll Str. 8, 07745-Jena, Germany; ^7^Department for Evolutionary Ecology, Institute for Zoology, Johannes Gutenberg University Mainz, Johann-Joachim-Becher-Weg 13, 55128 Mainz, Germany, and; ^8^Invertebrates Zoology Section, National Museums of Kenya, P. O. Box 40658-00100, Museum Hill Rd., Nairobi, Kenya (lnjoroge@museums.or.ke)

**Keywords:** mosquito-borne disease, vector ecology, genetics, culicine, *Anopheles*

## Abstract

The Lake Baringo and Lake Victoria regions of Kenya are associated with high seroprevalence of mosquito-transmitted arboviruses. However, molecular identification of potential mosquito vector species, including morphologically identified ones, remains scarce. To estimate the diversity, abundance, and distribution of mosquito vectors on the mainland shores and adjacent inhabited islands in these regions, we collected and morphologically identified adult and immature mosquitoes and obtained the corresponding sequence variation at cytochrome c oxidase 1 (COI) and internal transcribed spacer region 2 (ITS2) gene regions. A total of 63 species (including five subspecies) were collected from both study areas, 47 of which have previously been implicated as disease vectors. Fourteen species were found only on island sites, which are rarely included in mosquito diversity surveys. We collected more mosquitoes, yet with lower species composition, at Lake Baringo (40,229 mosquitoes, 32 species) than at Lake Victoria (22,393 mosquitoes, 54 species). Phylogenetic analysis of COI gene sequences revealed *Culex perexiguus* and *Cx*. *tenagius* that could not be distinguished morphologically. Most *Culex* species clustered into a heterogeneous clade with closely related sequences, while *Culex pipiens* clustered into two distinct COI and ITS2 clades. These data suggest limitations in current morphological identification keys. This is the first DNA barcode report of Kenyan mosquitoes. To improve mosquito species identification, morphological identifications should be supported by their molecular data, while diversity surveys should target both adults and immatures. The diversity of native mosquito disease vectors identified in this study impacts disease transmission risks to humans and livestock.

Mosquitoes are important vectors of filarial worms, malaria parasites, and arboviruses that are endemic to sub-Saharan Africa (Mwandawiro et al. 1997, Mwangangi et al. 2013, Ochieng et al. 2013). Earlier studies in the Zika Forest of Uganda part of Lake Victoria (LV) reported the circulation of arboviruses such as Zika and Usutu viruses in *Aedes africanus* and *Coquillettidia aurites* mosquitoes, respectively (Haddow et al. 1964). Zika virus has since been implicated in dengue-like disease syndromes and fetal microencephaly cases in the South Pacific and South and Central Americas (Mlakar et al. 2016), with *Aedes aegypti* and *Aedes albopictus* as its main vectors (Chouin-Carneiro et al. 2016). However, most studies around Kenya’s LV and Lake Baringo (LB) have focused on malaria vectors because of malaria endemicity in these areas (Mala et al. 2011, Olanga et al. 2015). Recent Rift Valley fever (RVF) virus outbreaks near LB have led to greater emphasis on arbovirus mosquito vector surveillance during and after outbreaks (Sang et al. 2010, Ochieng et al. 2013). The mainland shores and islands of LV and LB have similar aquatic and terrestrial biogeographies with favorable tropical climates that support diverse mosquito species, such as *Culex* L., *Aedeomyia* Theobald, *Aedes* Meigen, *Mansonia* Blanchard, and *Anopheles* Meigen species (Ofulla et al. 2010, Olanga et al. 2015, Omondi et al. 2015) and *Coquillettidia* Dyar (Haddow et al. 1964), that are known vectors of disease pathogens. Despite the importance of mosquitoes to public health in these two lake regions, little is known about their species diversity and distribution along the shores and adjacent islands of LV and LB.

Previous studies on mosquito species composition around both Kenyan lakes were biased toward trapping only one developmental stage of the mosquitoes, either only adults (Chen et al. 2004, Lutomiah et al. 2013, Omondi et al. 2015) or immatures (Chen et al. 2006, Imbahale et al. 2011). Targeting more developmental stages during mosquito sampling surveys provides better description of mosquito species diversity, as demonstrated in the 1950s in a study on the Kenyan coast (van Someren et al. 1955). Indeed, studies that targeted both the adult and immature mosquitoes report more species diversity (Linthicum et al. 1985, Sang et al. 2008).

Previous mosquito diversity studies around both Kenyan lakes have mainly employed morphology to identify the different species (Lutomiah et al. 2013). In the LV basin, population genetic studies on the *Anopheles gambiae* Giles complex (Chen et al. 2004, 2006) reflects research concentration on malaria, while at LB, mitochondrial and nuclear gene studies on *Aedes mcintoshi* Huang highlight this vector’s importance in RVF virus transmission (Tchouassi et al. 2014). Some mosquito species are difficult to identify as adults when features such as legs or scales are lost (Edwards 1941). In addition, many mosquitoes exist as species complexes, such as *Culex pipiens* L. sensu lato (s.l.) (Cornel et al. 2012) and *Anopheles gambiae* s.l. (Scott et al. 1993), limiting their identification based on morphology alone. However, genetic analyses to support morphology provides better taxonomic elucidation of species diversity (Kumar et al. 2007), thereby unraveling insights into disease epidemiology driven by the population genetic structures of species and subspecies (Tchouassi et al. 2014). In other parts of the world, mosquitoes have been successfully differentiated using the mitochondrial cytochrome c oxidase 1 (COI) gene, also known as the DNA barcode gene (Hebert et al. 2003, Ashfaq et al. 2014), and the ribosomal internal spacer region (Sum et al. 2014).

This study sought to expand understanding of the population structures, genetic diversity, and abundance of mosquito species in Kenyan lake biogeographies, which have remained limited despite documented circulation of mosquito-transmitted human and livestock pathogens. We investigated mosquito species composition, distribution, and genetic diversity on islands and adjacent mainland shores of LV and LB in Kenya, targeting all mosquito stages (egg, larva, pupa, and adult). We combined morphological and sequence data to provide a robust assessment of the diversity of key disease vectors in western Kenya.

## Materials and Methods

### Study Areas

**Fig. 1.**
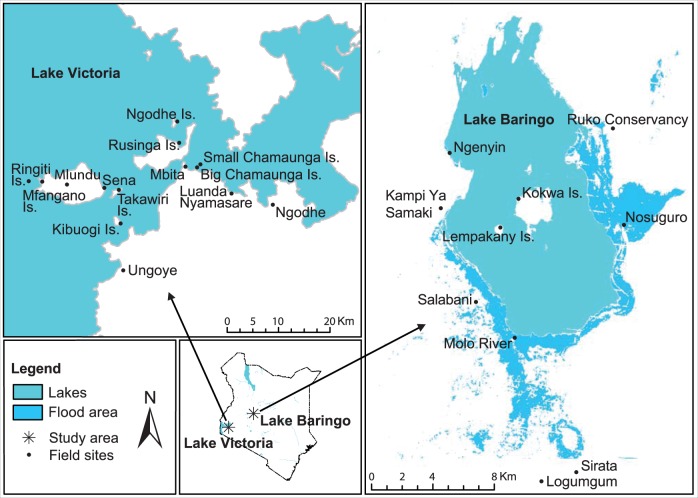
Map showing the sampling sites in two study areas in Kenya.

The study was carried out along the shores and adjacent islands of LV (Homa Bay County) and LB (Baringo County) in Kenya (Fig. 1). Ecologically, the islands and mainland shores of LV consist mainly of bushland, while those of LB are characterized by shrubs and grassland vegetation, with flooding that occurs during the rainy seasons (April–August, October–November; Johansson and Svensson 2002), transforming the landscape into marshland. We measured the weather conditions (wind speed, temperature, relative humidity, and barometric pressure) of each study site at the time of sampling using the Kestrel 4500 Pocket Weather Tracker (Nielsen-Kellerman, USA) in May and November at LV sites and in July and October at LB sites.

At LV in Homa Bay County, mosquitoes were sampled from four mainland shore sites in (Mbita, Luanda Nyamasare, Ngodhe, and Ungoye) and seven ecologically distinct islands (Chamaunga, Kibuogi, Takawiri, Mfangano, Ringiti, Rusinga, and Ngodhe; Fig. 1). Most of the inhabitants are ethnic Luo and Suba fishermen and small-scale farmers. Mbita is a mainland urban center that is connected to Rusinga Island *via* a causeway. The nearby twin Chamaunga Islands (referred to as “Big” and “Small”) are ∼500 m apart, with no permanent human habitation except for fishermen who occasionally use them as operating stations. Common animals found in these twin islands are the hippopotami and monitor lizards. Ringiti Island is rocky and inhabited by fishermen who have built aluminum sheet houses. The terrain in Ngodhe and Kibuogi Islands is mainly sloping, making the ground unable to hold stagnant water. Rusinga and Mfangano Islands are the largest and have significant human habitation of ∼43,000 people collectively (Opiyo et al. 2007, Masiwo 2015). On Mfangano Island, sampling was done in three locations (Milundu, Soklo, and Sena).

Lake Baringo is located in Baringo County in the Great Rift Valley (Fig. 1). Mosquitoes were sampled from Molo River, six mainland towns (Kampi ya Samaki, Ngenyin, Sengekeyon (Ruko Conservancy), Salabani, Logumgum, and Sirata), and three islands (Lempakany, Kokwa, and Nosuguro). Molo River was sampled at its point of entry into LB (Fig. 1). The water level of LB has been constantly rising in the recent past and thus, the lake is exceeding its borders (Onywere et al. 2013). The inhabitants of Baringo County are mostly livestock keepers with small-scale crop production and fishing.

### Mosquito Sampling

Mosquito samples were collected during the wet seasons of 2012 (LB: two-night pilot study between March 2–4, subsequent sampling between July 16–24 and between October 12–21; LV: April 2–15, May 18–31, and November 12–29). Adult mosquitoes were collected using CO_2_-baited CDC light traps (CDC Lt; John W. Hock Company, Gainesville, FL) targeting night-biting mosquito species, BG sentinel trap with its lure (Biogents, Regensburg, Germany) for trapping day-biting *Aedes* species, and Mosquito Magnet-X (MM-X) trap (American Biophysics Cooperation, North Kingstown, RI) with Mbita Blend (Mukabana et al. 2012) for trapping *Anopheles gambiae*. The CDC Lt (7 at LB and 13 at LV) and MMX traps (4 at LV) were set every evening from 18:00 h and retrieved from 07:00 h the following morning at each site. Lake Baringo mosquitoes were trapped over 126 trap nights, using three CDC Lts per night during each of the two pilot study sites and an average of seven CDC Lts per night set once at each of the 10 sites for two nights (one night per sampling month). Lake Victoria had 429 trap nights, comprising an average of 13 CDC Lts that were set in only one of the 11 sites per night for three nights (one night per sampling month).

Each BG sentinel trap was set from 06:30 and removed by 17:30. Locally designed oviposition traps (ovitraps) were created by cutting plastic water bottles (2 l) into two, filling each half to three-quarter with fresh water (750 ml), submerging oviposition paper all round the inside of the container and wrapping the outside completely with black waterproof bags to create a dark interior. Ten of these were then placed either on the ground and trees, or half-buried in the ground in the field, left for 7 days at each site to trap mosquito eggs, monitored daily and water was added to ovitraps with less than one-quarter level of water.

Standard mosquito dippers (Dipper; 350 ml; Bioquip Products, Rancho Dominguez, CA) with wooden handles were used to collect immature mosquitoes (eggs, larvae, and pupae) from their natural breeding sites such as ground water pools, rock holes, and tree holes by dipping three to five times per breeding site. We sampled immature mosquitoes from 25 and 138 breeding sites in LB and LV areas, respectively. The LB sites included 21 sampling sites on the mainland (14 in Kampi ya Samaki, 4 in Ruko, 2 in Sirata, and 1 in Salabani) and 4 on the islands (1 in Nosuguro and 3 in Kokwa Island). Around LV, immature mosquitoes were collected from 43 breeding sites on the mainland (3 in Luanda Nyamasare, 31 in Mbita, 3 in Ngodhe, and 6 in Ungoye) and 95 on the islands (15 in Chamaunga Island, 7 in Kibuogi Island, 36 in Mfangano Island, 6 in Ngodhe Island, 1 in Ringiti Island, 25 in Rusinga Island, and 5 in Takawiri Island).

### Morphological Identification and Mosquito Rearing

The immature stages of the mosquitoes (eggs, larvae, and pupae) were reared in their field-collected water to the adult stage in the *icipe* insectary under established protocols (Gerberg et al. 1994, Das et al. 2007). All adult mosquitoes were identified and sorted into species using standard morphological keys (Edwards 1941, Gillies and Meillon 1968, Gillett 1972, Gillies and Coetzee 1987, Jupp 1996). Mosquito species names were assigned according to Gillies and Meillon (1968) and Gillies and Coetzee (1987) for anophelines, and Edwards (1941) and Jupp (1996) for culicines. The adult mosquitoes were stored in 1.5-ml microcentrifuge tubes at −80°C until further analyses.

### Molecular Identification of Mosquitoes

We extracted genomic DNA from one leg of individual mosquitoes following the hot Sodium Hydroxide and Tris (HotSHOT) protocol (Montero-Pau et al. 2008). Briefly, one mosquito leg was put in 30 µl of alkaline lysis buffer (25 mM NaOH, 0.2 mM disodium EDTA, pH 8.0) and incubated in a thermocycler at 95°C for 30 min and cooled at 4°C for 5 min. Then, 30 µl neutralizing solution (40 mM Tris-HCl) was added. The resulting DNA was stored at −20°C until required as template for polymerase chain reaction (PCR) assays. Each PCR reaction was done using Hot Start Phusion kit (Thermo Scientific, Waltham, MA) and contained 4 µl of HF buffer, 0.4 µl of 10 mM dNTP mix, 1 µl of 10 mM primers, 0.6 µl DMSO, 1 µl of DNA, and distilled water to form a final volume of 20 µl. The mitochondrial cytochrome c oxidase 1 (COI) gene was amplified using forward (LCO1490 GGTCAACAAATCATAAAGATATTGG) and reverse (HCO2198 TAAACTTCAGGGTGACCAAAAAATCA) primers (Folmer et al. 1994) targeting the DNA barcode region or using LCO1490 forward and TL2-N-3014 TCCAATGCACTAATCTGCCATATTA (Simon et al. 1994) reverse primers targeting the longer fragment of more than 1,000 bases. The thermal cycling condition included a 2-min initial denaturation step at 98°C followed by 40 cycles of 10-s at 98°C for denaturation, 30-s annealing at 50°C and 40-s elongation at 72°C, and a final elongation at 72°C for 7-min. Also, 1,500 base pairs of the ribosomal internal transcribed spacer 2 (ITS2) region was amplified using CAS18sF1 TACACACCGCCCGTCGCTACTA forward (Ji et al. 2003) and ITS2 porter28s ATGCTTAAATTTAGGGGGTAGTC reverse (Cornel et al. 2012) primers. The thermal cycling condition included a 30-s initial denaturation step at 98°C followed by 35 cycles of 10-s at 98°C for denaturation, 30-s annealing at 58°C and 40-s elongation at 72°C, and a final elongation step of 72°C for 7-min. Finally, *Anopheles gambiae* complex mosquitoes were identified using the ribosomal DNA-PCR method as described by Scott (1993). All successful amplifications with single bands of expected size, as confirmed using 1.5% agarose gel electrophoresis, were purified using the ExoSAP-IT for PCR Product Kit (Affymetrix Inc., Santa Clara, CA) and sent to Macrogen (South Korea) for Sanger sequencing.

Individuals of 29 mosquito species from the two lake sites were digitally photographed. One leg from each specimen was placed into a 96-well plate containing 30 µl of 95% ethanol and sent to the Canadian Centre for DNA Barcoding (CCDB) in Canada for DNA amplification and sequencing of the COI gene using cocktail primers C_LepFolF (cocktail of LepF1 and LCOI490) and C_LepFolR (cocktail of LepR1 and HCO2198; Ivanova and Grainger 2006). Each digital mosquito image was named with sample IDs corresponding to specific positions in the 96-well plate and submitted to the Barcode of Life Database (BOLD; Ratnasingham and Hebert 2007) along with taxonomic and collection data, and voucher and specimen details. The CCDB also edited the sequences and uploaded the resulting data on the BOLD website.

### Data Analyses

Mosquito abundance data collected from traps were analyzed using generalized linear model (GLM) with log link and negative binomial distribution error to examine differences between LV and LB, and between mainland and island within the areas. The GLM models were fitted to the most dominant genera (*Aedes*, *Culex*, *Anopheles*, and *Mansonia*). Incident rate ratio (IRR) and 95% confidence intervals for the IRR were estimated from the GLM model. The analyses were performed in R 3.2.1 (R Core Team 2015). The wind speed data were classified using the wind speed table on the Windfinder webpage (http://www.windfinder.com/wind/windspeed.htm). Relative abundances of mosquitoes were calculated as the mean numbers of adult mosquitoes collected per trap and immature mosquitoes collected per dip from the island or mainland sites in each sampling month.

#### DNA Sequence Analyses

The COI and ITS2 mosquito DNA sequences were edited in Geneious R7.1.9 software (Kearse et al. 2012). After trimming, sequences <200 bases were excluded from the analyses and all remaining sequences were compared to reference sequences on the BOLD (COI sequences only; Ratnasingham and Hebert 2007) and GenBank (Benson et al. 2014) databases. The barcode index number (BIN) system (Ratnasingham and Hebert 2013) in BOLD was used to identify mosquito COI sequences belonging to similar taxonomic clusters, while the “barcode gap analyses” in BOLD was used to analyze intraspecific and interspecific sequence divergence of submitted sequences. The DNA sequences that matched those of mosquitoes from the two databases were further analyzed. Multiple sequence alignments were generated using MAFFT (Katoh et al. 2002) v7.017 plugin in Geneious R7.1.9 software (Biomatters, San Francisco, CA). Phylogenetic analyses on the resultant DNA alignments were done with Randomized Axelerated Maximum Likelihood (RAxML) version 8.2.0 (Stamatakis 2014) using 1,000 rapid-bootstrapping (Felsenstein 1985) and subsequent Maximum Likelihood (ML) search and general time reversible (GTR) model with the gamma model of rate heterogeneity option. The output tree having the best-scoring ML with bootstrap support values was edited with midpoint rooting and depicted using FigTree software version 1.4.2 (Rambaut 2014).

## Results

### Mosquito Diversity

**Fig. 2.**
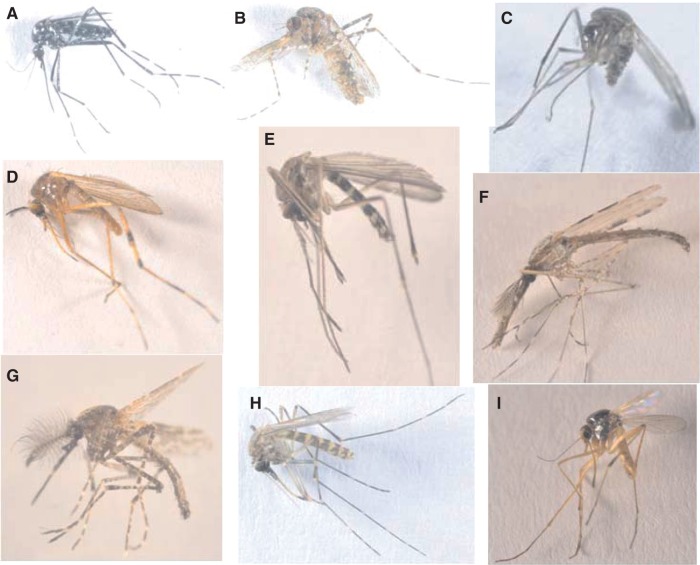
Photographs of the dorsal view of mosquito species representing the seven genera collected from Lake Victoria and Lake Baringo areas. (**A**) *Aedes* (*Stegomyia*) *metallicus*. (**B**) *Mansonia* (*Mansonoides*) *africana*. (**C**) *Culex* (*Neoculex*) *rima*. (**D**) *Coquillettidia* (*Coquillettidia*) *microannulata*. (**E**) *Culex* (*Culex*) *watti*. (**F**) *Anopheles* (*Cellia*) *pharoensis*. (**G**) *Aedeomyia* (*Aedeomyia*) *africana*. (**H**) *Culex* (*Culex*) *duttoni*. (**I**) *Mimomyia* (*Mimomyia*) *splendens*. *Cx*. *rima* has not been reported in Kenya. *Cx*. *watti* and *Cx*. *duttoni* look morphologically similar and can be misidentified as each other.

**Fig. 3.**
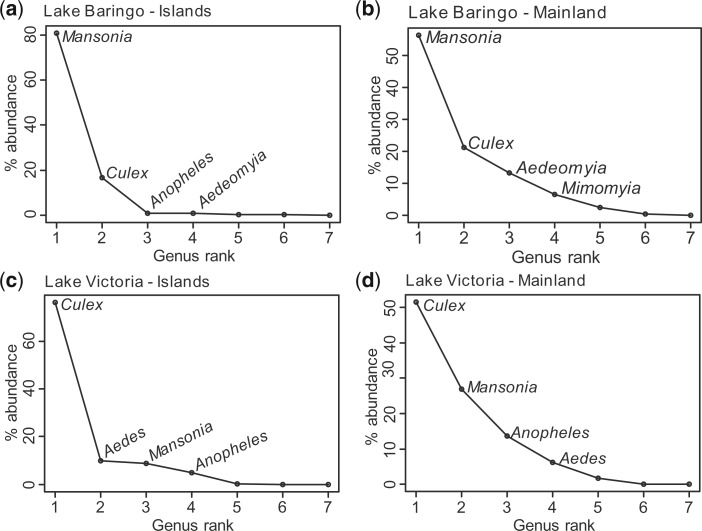
Rank abundance curves of mosquito genera from the two study areas: Lake Baringo (**a**) islands and (**b**) mainland towns; Lake Victoria (**c**) islands and (**d**) mainland towns.

A total of 62,622 mosquitoes (Table 1) comprising 63 species and subspecies (Tables 2–6) were collected and identified from the two study areas. Sixty-one of these species were identified morphologically. The remaining two species were only identified through molecular methods using sequence data (Table 2). Of this total, 40,229 (64.2%) were collected near LB and 22,393 (35.8%) were collected near LV (Table 1). Although the number of mosquitoes collected at LB was higher than that from LV, the number of mosquito species was lower (32 species) than at LV (54 species). Six mosquito genera (*Aedes*, *Anopheles*, *Mimomyia* Theobald, *Coquillettidia*, *Culex*, and *Mansonia*) were collected from both LB and LV areas (Table 1; Fig. 2). One genus, *Aedeomyia*, was found only at LB (Table 1; Fig. 3). It was not possible to identify some damaged specimens to species level and are presented as *Aedes* spp., *Anopheles* spp., *Culex* spp., *Culex* (*Neoculex*) spp., *Coquillettidia* spp., and *Mansonia* spp. Ten and 23 mosquito species collected from LB and LV environs, respectively, have not been reported in these regions before.

**Table 1.** Total mosquito genera composition collected per site using adult and immature sampling types per area: island versus mainland sites

Genus	Lake Baringo			Lake victoria			Grand total
	Island	Mainland	Total	Island	Mainland	Total	
*Aedeomyia* Theobald	51	5,009	5,060	0	0	0	5,060
*Aedes* Meigen	1	11	12	1,163	662	1,825	1,837
*Anopheles* Meigen	26	878	904	571	1,461	2,032	2,936
*Coquillettidia* Dyar	7	150	157	8	11	19	176
*Culex* L.	510	7,840	8,350	8,910	5,512	14,422	22,772
*Mansonia* Blanchard	2,472	20,835	23,307	1,013	2,889	3,902	27,209
*Mimomyia* Theobald	17	2,422	2,439	2	191	193	2,632
Total	3,084	37,145	40,229	11,667	10,726	22,393	62,622

**Table 2.** *Culex* species composition collected from island and mainland sites per study area using adult or immature trap types

Subgenus	Mosquito species	Trap	Lake Baringo			Lake victoria		
			Island	Mainland	Total	Island	Mainland	Total
Culex	*Cx. annulioris* Theobald	Dipper*^a^*	0	0	0	8	0	8
	*Cx. antennatus* (Becker)*^b^*	CDC Lt*^c^*	161	984	1,145	0	153	153
	*Cx. bitaeniorhynchus* Giles	CDC Lt*^c^*	0	5	5	0	0	0
	*Cx. duttoni* Theobald*^d^*	CDC Lt	0	0	0	3	0	3
		Dipper	0	2	2	1	12	13
	*Cx. ethiopicus* Edwards	CDC Lt*^c^*	0	2	2	0	0	0
	*Cx. neavei* Theobald*^b^*	CDC Lt	0	1	1	1,299	65	1,364
		Dipper	0	9	9	13	5	18
	*Cx. perexiguus* Theobald*^b^**^,^**^d^*	Dipper*^a^*	0	4	4	1	0	1
	*Cx. pipiens* L.	CDC Lt	25	1,304	1,329	568	2,796	3,364
		Dipper	8	331	339	1,119	302	1,421
	*Cx. poicilipes* Theobald	CDC Lt	187	4,903	5,090	34	15	49
		Dipper	0	3	3	88	1	89
	*Cx. simpsoni* Theobald*^d^*	Dipper*^a^*	0	0	0	18	9	27
	*Cx. sinaiticus* Kirkpatrick*^d^*	CDC Lt*^c^*	0	0	0	8	0	8
	*Cx. striatipes* Edwards*^b^**^,^**^d^*	CDC Lt	0	22	22	26	174	200
		Dipper	0	0	0	3	2	5
	*Cx. tenagius* Van Someren*^b^*	CDC Lt*^c^*	0	1	1	0	0	0
	*Cx. terzii* Edwards*^d^*	Dipper*^a^*	0	0	0	2	0	2
	*Cx. thalassius* Theobald*^b^**^,^**^d^*	CDC Lt*^c^*	0	1	1	10	0	10
	*Cx. theileri* Theobald*^d^*	Dipper*^a^*	0	0	0	1	1	2
	*Cx. univittatus* Theobald	CDC Lt	22	43	65	159	1,571	1,730
		Dipper	3	36	39	37	28	65
	*Cx. vansomereni* Edwards	CDC Lt	0	0	0	5	8	13
		Dipper	0	0	0	8	3	11
	*Cx. watti* Edwards*^b^**^,^**^d^*	Dipper*^a^*	0	1	1	30	19	49
	*Cx. zombaensis* Theobald	CDC Lt	0	0	0	1	16	17
		Dipper	0	5	5	14	0	14
Culiciomyia	*Cx. nebulosus* Theobald	CDC Lt*^c^*	0	0	0	4,773	2	4,775
Lutzia	*Cx. (Lutzia) tigripes* De Grandpre and De Charmoy	CDC Lt	0	0	0	25	0	25
		Dipper	0	3	3	20	6	26
Neoculex	*Cx. adersianus* Edwards*^b^*	Dipper*^a^*	0	0	0	10	0	10
	*Cx. rima* Theobald*^b^*	Dipper*^a^*	0	0	0	25	0	25
	*Cx. (Neoculex)* spp. Dyar	Dipper*^a^*	0	2	2	13	2	15
	*Culex* spp	CDC Lt	103	162	265	351	149	500
		Dipper	1	16	17	237	173	410
Total			510	7,840	8,350	8,910	5,512	14,422

*Cx*. *perexiguus* and *Cx*. *tenagius* were identified molecularly only.

*^a^* Mosquito samples captured using Dipper only.

*^b^* Mosquito species not previously reported in Lake Baringo.

*^c^* Mosquito samples captured using CDC Lt only.

*^d^* Mosquito species not previously reported in Lake Victoria.

Dipper, standard dipper; CDC Lt, CDC light trap.

At LB, *Mansonia* was the most abundant mosquito genus, accounting for 57.9% of the total samples collected (Fig. 3a and b), whereas *Aedes* ranked least with 12 specimens (0.03%). However, in the LV area, *Culex* (64.4%) was the most abundant genus (Fig. 3c and d), while *Coquillettidia* (0.1%) was least abundant.

Overall, the *Culex* genus had the highest number (24) of species collected (Table 2), belonging to four subgenera (*Culex*, *Culiciomyia* Theobald, *Neoculex* Dyar, *Lutzia* Theobald). This was, followed by 13 *Aedes* species from four subgenera (Table 3), 12 *Anopheles* species (including 5 subspecies) (Table 4), seven *Coquillettidia* species, two *Mansonia* species (Table 5), three *Mimomyia* species from two subgenera (Table 6), and two *Aedeomyia* species (Table 6).

**Table 3.** *Aedes* species composition collected from island and mainland sites per study area using adult or immature trap types

*Aedes* subgenus	Species	Trap	Lake Baringo			Lake Victoria		
			Island	Mainland	Total	Island	Mainland	Total
*Stegomyia*	*Ae. aegypti* (L.)	CDC Lt	0	0	0	11	1	12
		Dipper	0	0	0	479	261	740
	*Ae. luteocephalus* (Newstead)*^a^*	Dipper*^b^*	0	0	0	50	23	73
	*Ae. metallicus*(Edwards)	CDC Lt	0	0	0	5	5	10
		Dipper	0	0	0	73	209	282
	*Ae. simpsoni* (Theobald)*^a^*	CDC Lt*^c^*	0	0	0	5	0	5
	*Ae. vittatus* (Bigot)*^a^*	CDC Lt	0	0	0	49	4	53
		Dipper	0	0	0	303	0	303
*Aedimorphus*	*Ae. hirsutus*(Theobald)*^d^*	CDC Lt	1	0	1	18	16	34
		Dipper	0	0	0	7	0	7
	*Ae. cumminsi* (Theobald)	CDC Lt	0	0	0	21	6	27
		Dipper	0	0	0	17	0	17
	*Ae. dentatus* (Theobald)*^a^*	Dipper*^b^*	0	0	0	1	0	1
	*Ae. ochraceus* (Theobald)	CDC Lt	0	0	0	1	0	1
		Dipper	0	0	0	1	0	1
	*Ae. tarsalis* (Newstead)	CDC Lt*^c^*	0	0	0	0	31	31
*Diceromyia*	*Ae. furcifer* (Edwards)*^a^*	Dipper*^b^*	0	0	0	0	3	3
*Neomelanoconion*	*Ae. circumluteolus* (Theobald)	CDC Lt*^c^*	0	0	0	0	2	2
	*Ae. mcintoshi* Huang	CDC Lt*^c^*	0	0	0	3	0	3
	Ae. spp.	CDC Lt	0	11	11	19	12	31
		Dipper	0	0	0	100	89	189
**Totals**			1	11	12	1,163	662	1,825

*^a^* Mosquito species not previously reported in Lake Victoria.

*^b^* Mosquito samples captured using Dipper only.

*^c^* Mosquito samples captured using CDC Lt only.

*^d^* Mosquito species not previously reported in Lake Baringo.

Dipper, standard dipper; CDC Lt, CDC light trap.

**Table 4.** *Anopheles* species composition collected from island and mainland sites per study area using adult or immature trap types

Subgenus	Species	Trap	Lake Baringo			Lake Victoria		
			Island	Mainland	Total	Island	Mainland	Total
*Cellia*	*An. funestus* Giles	CDC Lt	1	166	167	44	24	68
		Dipper	0	0	0	2	1	3
	*An. gambiae* Giles s.l.	CDC Lt	0	20	20	58	1,073	71
		Dipper	0	38	38	227	49	276
	*An. pharoensis* Theobald	CDC Lt	12	84	96	0	7	7
		Dipper	0	12	12	0	0	283
	*An. squamosus* Theobald*^a^*	CDC Lt*^b^*	0	1	1	0	13	13
	*An. rufipes* (Gough)*^a^*	Dipper*^c^*	0	0	0	2	0	2
	*An. rhodesiensis* Theobald*^a^*	CDC Lt*^b^*	0	0	0	0	4	15
*Anopheles*	*An. coustani* Laveran complex	CDC Lt	9	431	440	174	254	428
		Dipper	0	0	0	1	0	1
	*An. symesi* Edwards*^a^**^,^**^d^*	CDC Lt*^b^*	0	0	0	1	0	429
	*An. tenebrosus* Donitz*^a^**^,^**^d^*	CDC Lt*^b^*	0	0	0	12	11	23
	*An. ziemanni* Grunberg*^a^**^,^**^d^*	CDC Lt	3	119	122	38	17	55
		Dipper	0	0	0	2	0	78
	*Anopheles* spp.	CDC Lt*^b^*	1	7	8	10	8	18
Total			26	878	904	571	1,461	2,032

*An*. *gambiae* s.l. contains *An*. *gambiae* s.s. and *An*. *arabiensis*.

*^a^* Mosquito species not previously reported in Lake Victoria.

*^b^* Mosquito samples captured using CDC Lt only.

*^c^* Mosquito samples captured using Dipper only.

*^d^* Members of the *An*. *coustani* complex.

Dipper, standard dipper; CDC Lt, CDC light trap.

**Table 5.** *Coquillettidia* and *Mansonia* species composition collected from island and mainland sites per study area using CDC light trap only

Subgenus	Species	Lake Baringo			Lake Victoria		
		Island	Mainland	Total	Island	Mainland	Total
*Coquillettidia*	*Cq. aurites* Theobald	4	10	14	0	1	1
	*Cq. chrysosoma* (Edwards)*^a^*	0	1	1	0	0	0
	*Cq. fuscopennata* (Theobald)	3	7	10	0	0	0
	*Cq. metallica* (Theobald)	0	132	132	0	0	0
	*Cq. microannulata* (Theobald)*^b^*	0	0	0	8	0	8
	*Cq. pseudoconopas* Theobald	0	0	0	0	1	1
	*Cq. versicolor* Edwards	0	0	0	0	7	7
	*Coquillettidia* spp.	0	0	0	0	2	2
*Mansonioides*	*Ma. africana* (Theobald)	1,055	18,918	19,973	787	1,571	2,358
	*Ma. uniformis* (Theobald)	398	1,291	1,689	202	1,118	1,320
	*Mansonia* spp.	1,019	626	1,645	24	200	224
Total		2,479	20,985	23,464	1,021	2,900	3,921

*^a^* Mosquito species not previously reported in Lake Baringo.

*^b^* Mosquito species not previously reported in Lake Victoria.

**Table 6.** *Aedeomyia* and *Mimomyia* species composition collected from island and mainland sites per study area using adult or immature trap types

Subgenus	Species	Trap	Lake Baringo			Lake Victoria		
			Island	Mainland	Total	Island	Mainland	Total
*Aedeomyia*	*Ad. africana* Neveu-Lemaire	CDC Lt*^a^*	26	4,911	4,937	0	0	0
*Lepiothauma*	*Ad. furfurea* (Enderlein)	CDC Lt*^a^*	25	98	123	0	0	0
*Mimomyia*	*Mi. hispida* (Theobald)	CDC Lt*^a^*	0	0	0	0	1	1
	*Mi. splendens* (Theobald)	CDC Lt	9	2,415	2,424	2	188	190
		Dipper	4	7	11	0	1	1
*Etorleptiomyia*	*Mi. mediolineata* (Theobald)*^b^*	CDC Lt*^a^*	4	0	4	0	0	0
Total			68	7,431	7,499	2	190	192

*^a^* Mosquito samples captured using CDC Lt only.

*^b^* Mosquito species not previously reported in Lake Baringo.

Dipper, standard dipper; CDC Lt, CDC light trap.

At species level, *Mansonia africana* (Theobald) (Fig. 2B) was the most abundant in Baringo (19,973) while *Culex* (*Culex*) *pipiens* was the most abundant at LV (4,785). Only single specimens of *Cx*. *tenagius* van Someren, *Cx*. (*Cux*.) *watti* Edwards (Fig. 2E), *Ae*. (*Aedimorphus*) *hirsutus* (Theobald), *An*. (*Cellia*) *squamosus* Theobald, *Cx*. (*Cux*.) *thalassius* Theobald, and *Cq*. *chrysosoma* (Edwards) were collected at LB. At LV, the least collected mosquitoes included single specimens of *Cq*. *aurites* Theobald, *Ae*. (*Adm*.) *dentatus* (Theobald), *Cq*. *pseudoconopas* Theobald, *Cx*. *perexiguus* Theobald, *Mi*. (*Mimomyia*) *hispida* (Theobald), *An*. (*Anopheles*) *symesi* Edwards (a member of the *An*. (*Ano*.) *coustani* Laveran complex), and *An*. (*Cel*.) *rivulorum* Leeson (a member of the *An*. (*Cel*.) *funestus* Giles group).

Morphologically, species of the *An*. *coustani* complex were identified based on their hind legs (Gillett 1972) and specimens with damaged hind legs were labeled as *An*. *coustani* complex (Table 4). Similarly, members of the *An*. *funestus* group that could not be identified to species level were left as *sensu lato* (s.l.) (Table 4). Only one of the three members of the *An*. *coustani* complex (*An*. (*Ano*.) *ziemanni* Grünberg) was obtained from LB, whereas three members (*An*. *symesi*, *An*. *tenebrosus* Donitz, and *An*. *ziemanni*) were found at LV. A single member of *An*. *funestus* group, *An. rivulorum*, was found at LV. Out of the 137 specimens of *Anopheles gambiae* s.l. molecularly analyzed, three (2.2%) were *An*. *gambiae* sensu stricto (s.s.) found in LV sites only, 119 (86.9%) were *An*. *arabiensis* Patton, while 15 (10.9%) specimens failed to amplify by PCR.

### Molecular Versus Morphological Identification

**Fig. 4.**
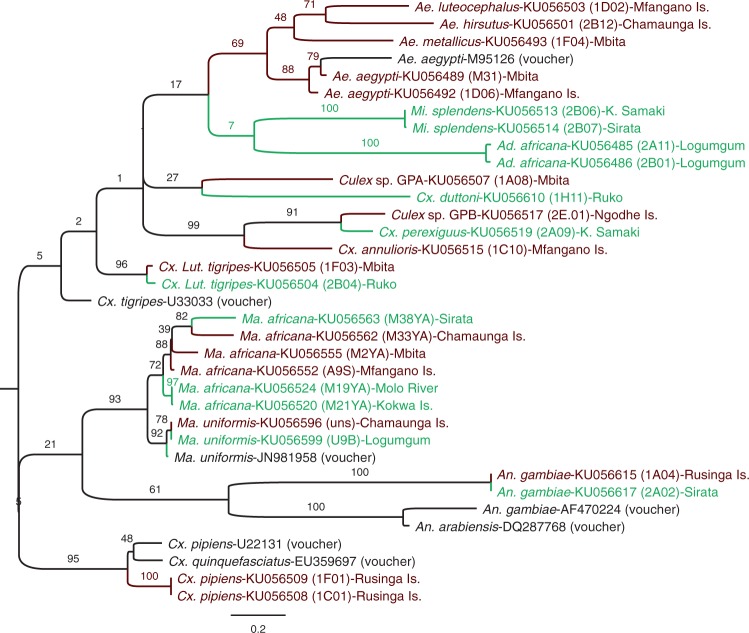
ITS2 gene sequence Maximum Likelihood phylogenetic tree of selected mosquito species from Lake Victoria and Lake Baringo regions of Kenya. Names in black containing “voucher” are sequences included in the analyses from GenBank with their accession numbers. Taxon names in green are from Lake Baringo sites, and those in red are from Lake Victoria sites. The taxa are labeled with name of mosquito species, then GenBank Accession number, with the sequence ID in brackets and the exact site location at the end. Sites ending with “Is.” are Island sites, K. Samaki is Kampi ya Samaki.

**Fig. 5.**
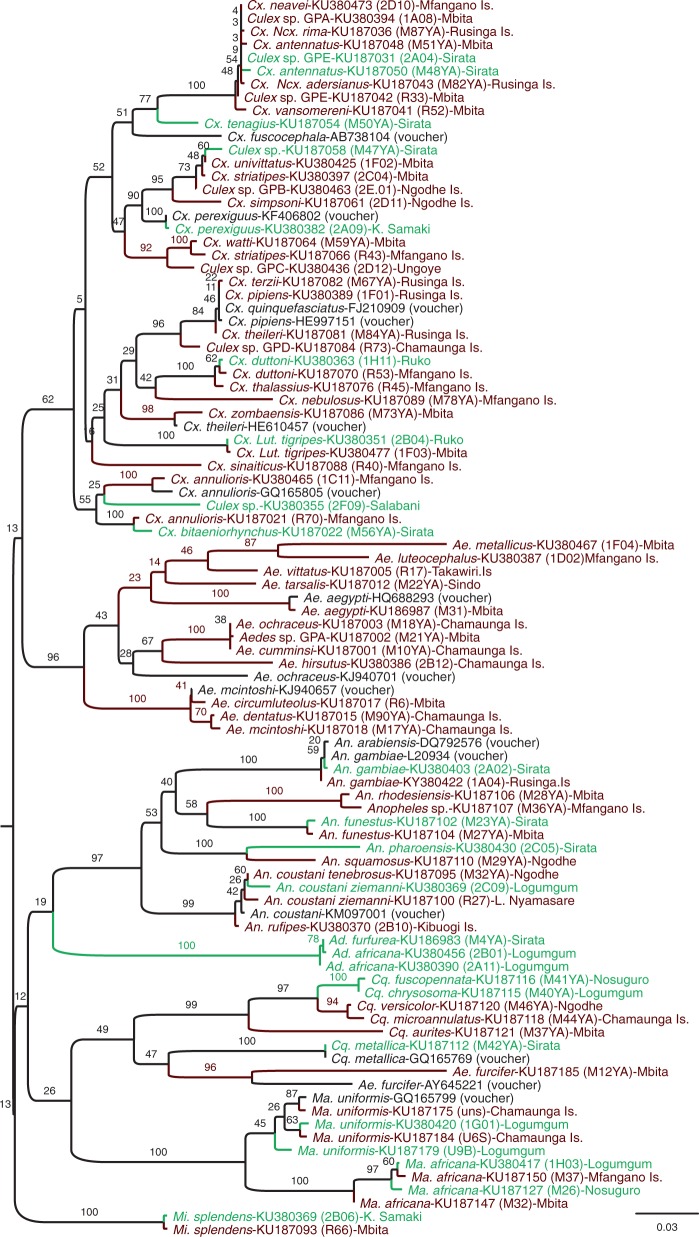
COI gene sequence Maximum Likelihood phylogenetic tree of selected mosquito species from Lake Victoria and Lake Baringo regions of Kenya. Names in black containing “voucher” are sequences included in the analyses from GenBank with their accession numbers. Taxon names in green are from Lake Baringo sites, and those in red are from Lake Victoria sites. The taxa are labeled with name of mosquito species, then GenBank Accession number, with the sequence ID in brackets and the exact site location at the end. Sites ending with “Is.” are Island sites, K. Samaki is Kampi ya Samaki, L. Nyamasare is Luanda Nyamasare.

A total of 341 COI gene (Supp. Table 1 [online only]) and 133 ITS2 gene (Supp. Table 2 [online only]) sequences belonging to 54 and 14 mosquito species, respectively, were successfully generated and submitted to GenBank (ITS2 sequence accession numbers: KU056485–KU056617; COI sequence accession numbers: KU186979–KU187186). Out of these, 133 samples (GenBank accession numbers: KU380347–KU380479) of 29 mosquito species from different sampling sites (Supp. Table 1 [online only]) have accompanying photographs available on BOLD from the DS-KMOSQBV dataset. Maximum likelihood phylogenetic trees of the ITS2 (Fig. 4, Supp. Fig. 1 [online only]) and COI (Fig. 5, Supp. Fig. 2 [online only]) gene sequences concurred in clustering patterns among mosquito genera and species. Twenty-six ITS2 samples did not have corresponding COI sequences and 234 COI samples did not have corresponding ITS2 sequences, as these samples failed to amplify by PCR. However, 107 ITS2 sequences have complimentary COI sequences as indicated by their sample IDs on the phylogenetic trees. COI gene sequences isolated from morphologically identified mosquito species confirmed the identity of 52 species and uncovered two additional species (*Cx*. *perexiguus*, *Cx*. *tenagius*) that were morphologically misidentified. Specifically, morphologically identified specimens of *Cx. univittatus* were detected by the BOLD BIN system as *Cx. perexiguus* (BOLD:AAM3892), a close species to *Cx*. *univittatus*, but differentiated via the male genitalia. Specimens of *Cx*. *antennatus* were similarly corrected to *Cx*. *tenagius*.

Some specimens morphologically identified to the species level, could only be resolved to the genus level based on their COI sequences and five closely related specimens that were misidentified were resolved to the species level based on their COI sequences. Morphologically misidentified male samples of *Ae*. *dendrophilus keniensis* and *Ma*. *uniformis* were corrected to *Ae*. *metallicus* (BOLD:ACK2517) and *Ma*. *africana*, respectively, based on their COI sequences. Similarly, as *Culex watti* (Fig. 2E) and *Cx. duttoni* (Fig. 2H) are morphologically similar, a sample of *Cx*. *watti* was re-designated as *Cx*. *duttoni*, a sample of *Cx*. *duttoni* was changed to *Cx*. *watti*. Additionally, a *Culex* sp*.* sample resembled *Cx*. *pipiens* but had lower mesepimeral bristles (Edwards 1941), and was initially recorded as *Cx*. *watti*. Based on COI sequence analysis, four samples could only be identified to the genus level, as they did not match any available mosquito species on GenBank or BOLD and were therefore reported as *Anopheles* sp., *Culex* sp. GP-B (BOLD:AAY8999), *Culex* sp. GP-C (BOLD:ACK8634), and *Culex* sp. GP-D. Sequences of *Cx*. *pipiens* fell into two separate BINs, that contained either *Cx*. *pipiens* (BOLD:AAA4751) mosquitoes or many different *Culex* species that are probably understudied, therefore renamed to *Culex* sp. GP-A and *Culex* sp. GP-E (BOLD:AAT8827). Further, the two BINs formed two clusters on the phylogenetic trees (Figs. 4 and 5, Supp. Figs. 1 and 2 [online only]). However, *Aedeomyia africana* and *Ad*. *furfurea* shared the same BIN (BOLD:ACK8488), with similar COI gene sequences clustering into a distinct clade. However, the ITS2 gene region failed to amplify to confirm the *Aedeomyia* species. Though recognized as a genus (Tanaka 2003), the *Lutzia* ITS and COI sequences clustered within the *Culex* clade. A clade of *Culex* mosquitoes consisted of different morphologically identified *Cx*. *pipiens* mosquitoes (reported as *Culex* sp. GP-A and *Culex* sp. GP-E), and *Cx*. *vansomereni*, *Cx*. *neavei*, *Cx*. (*Neoculex*) *rima*, and *Cx*. (*Ncx*.) *adersianus* without close COI sequence matches on GenBank (sequences were ≤95% identical to those on GenBank) or BOLD. This demonstrates the difficulty in the morphological identification of *Culex* mosquitoes, which look very similar and are sometimes differentiated by minor taxonomic characters that may be lost during trapping and handling.

*Mansonia africana* of LB formed distinct ITS2 (Fig. 4) and COI (Fig. 5) subclusters among the basal and more diverse *Ma*. *africana* sequences obtained from samples collected at LV, which were more divergent on the phylogenetic trees from each other than those in LB. A similar trend was evident in the COI phylogeny of *Ma. uniformis* mosquitoes, with LV populations being basal to most LB samples.

The *Aedes* sp. GP-A cluster close to *Ae*. *cumminsii* in the same clade with *Ae*. *ochraceus*, which is also a member of *Aedimorphus* subgenus of *Aedes*. The COI phylogeny revealed minimal sequence divergence among subspecies of the *Anopheles coustani* complex. Though they were identified to subspecies based on their hind legs, the COI barcode region could not differentiate them.

### Disease Vectors

Forty-seven (75.4%) mosquito species and subspecies in all the genera identified in this study are associated with disease pathogens (Supp. Table 3 [online only]). *Anopheles funestus* and *An*. *arabiensis* Patton are known vectors of human malaria parasites in LV area, whereas viruses have been isolated from *Aedeomyia africana* Neveu-Lemaire (Fig. 2G), *An*. *coustani*, *Coquillettidia fuscopennata* (Theobald), *Culex pipiens*, *Cx*. *univittatus* Theobald, *Mansonia africana*, and *Ma*. *uniformis* (Theobald) in LB area (Supp. Table 3 [online only]). Also, viruses have been isolated from 13 additional mosquito species belonging to the *Culex*, *Anopheles*, and *Aedes* genera in Kenya (Supp. Table 3 [online only]). The most abundant vector species in both study areas was *Mansonia*, followed by *Culex* and *Anopheles* species. *Aedes* vector species were only present at LV, while *Cq*. *fuscopennata* was present only at LB. Though not known vectors in Kenya, the other 25 mosquitoes are known to transmit diseases in other countries (Supp. Table 3 [online only]). Out of the 64 species sampled, we could not find any published disease associations for 16 species: *Ad*. *furfurea* (Enderlein), *An*. *symesi*, *An*. *rhodesiensis* Theobald, *Cq*. *chrysosoma*, *Cx*. *duttoni* Theobald (Fig. 2H), *Cx*. *nebulosus* Theobald, *Cx*. *simpsoni* Theobald, *Cx*. *sinaiticus* Kirkpatrick, *Cx*. *striatipes* Edwards, *Cx*. *tenagius*, *Cx*. *terzii* Edwards, *Cx*. *watti*, *Cx*. (*Lut*.) *tigripes* De Grandpre and De Charmoy, *Cx*. (*Ncx*.) *adersianus* Edwards, *Cx*. (*Ncx*.) *rima* Theobald (Fig. 2C), and *Mi*. *mediolineata* (Theobald).

### Comparison of Sampling Method Captures

Mosquito sampling techniques were grouped into CDC Lt for adult mosquitoes and Dipper for mosquito immatures (Tables 2–6). Most of the mosquitoes sampled were adults trapped using CDC Lt, which collected 58,025 specimens (49 species) comprising 92.7% of all specimens, while immature mosquitoes collected by Dipper represented 4,597 specimens (35 species) representing 7.3% of the total collection. Of these, only 21 species were common for both sampling methods. Neither larval nor other immature stage samples of *Mansonia* and *Aedeomyia* species were collected from either lake region.

Ovitraps set to collect the mosquito eggs did not collect any eggs despite repeated sampling and thus were discontinued. The use of the four MM-X traps was also discontinued because they trapped less than five mosquitoes and less than three genera per trapping period. At LV, *Aedes dentatus* (Theobald), *Ae*. *furcifer* (Edwards), *Ae*. *luteocephalus* (Newstead), *An*. *rufipes* (Gough), *Culex annulioris* Theobald, all *Culex* (*Neoculex*), *Cx*. *perexiguus*, *Cx*. *simpsoni*, *Cx*. *terzii*, *Cx*. *theileri*, and *Cx*. *watti* were sampled by Dipper only, while CDC Lt alone caught *Ae*. *circumluteolus*, *Ae*. *mcintoshi*, *Ae*. *simpsoni* (Theobald), *Ae*. *tarsalis* (Newstead), *Cx*. *antennatus*, *Cx*. *sinaiticus*, *Cx*. *thalassius*, *Cx*. *nebulosus*, *Mimomyia hispida*, all *Coquillettidia*, and *Anopheles* species (except *An*. *rufipes* which was collected only using Dipper). At LB, no *Aedes*, *Aedeomyia*, and *Coquillettidia* species were collected using the Dipper. *Cx*. *duttoni*, *Cx*. *watti*, *Cx*. *perexiguus*, *Cx*. *zombaensis*, *Cx*. *tigripes*, and *Cx*. (*Neoculex*) spp. were collected by Dipper only at LB. Also, *Aedeomyia furfurea*, *Ad*. *africana*, *Cx*. *antennatus*, *Cx*. *bitaeniorhynchus*, *Cx*. *ethiopicus* Edwards, *Cx*. *sinaiticus*, *Cx*. *tenagius*, *Cx*. *thalassius*, *Cq*. *chrysosoma*, *Cq*. *fuscopennata*, *Cq*. *metallica* (Theobald), and *Mi*. (*Etorleptiomyia*) *mediolineata* were collected only by CDC Lt at LB.

Further, the presence of two mosquito species, *Cx*. (*Culex*) *sinaiticus* (collected with CDC Lt only) and *Cx*. (*Neoculex*) *rima* (collected with Dipper only; Fig. 2C), which have never been reported in Kenya or as vector species, were found on the two big LV islands of Mfangano and Rusinga, respectively. A third species, *Cx*. *perexiguus*, reported for the first time in Kenya, was collected only with a Dipper at Kampi ya Samaki, a mainland site near LB and on Mfangano Island in LV.

### Island Versus Mainland Mosquito Collections

The proportion of mosquitoes at the mainland sites (76.4%) was higher than at island sites (23.6%; *n* = 62,622). Thirty-six mosquito species were found on both islands and mainland sites while 14 species were only found on islands and 13 only on the mainland sites. However, some species were found only in specific sites. For instance, *Aedeomyia africana* and *Ad*. *furfurea* were mostly found in Baringo mainland sites, and not at all at LV (Table 6).

The genus *Aedes* was more than one hundred times more abundant near LV area than near LB, χ^2 ^= 19.0, df = 1, *P* < 0.0001 (Table 3). Within LV, the difference in *Aedes* species abundance between the mainland and island was not significant, χ^2 ^= 0.19, df = 1, *P* = 0.996. The only *Ae*. *hirsutus* specimen sampled around LB was collected on Kokwa Island. Of the 13 *Aedes* species collected at LV, 4 species (*Ae*. *simpsoni*, *Ae*. *dentatus*, *Ae*. *ochraceus*, and *Ae*. *mcintoshi*) were exclusively collected from the islands, while 3 species (*Ae*. *tarsalis*, *Ae*. *furcifer*, and *Ae*. *circumluteolus*) were only collected from the mainland sites.

The abundance of *Anopheles* species was not significantly different between LV and LB. However, the mainland had significantly more catches and two extra species and subspecies (*An*. *arabiensis* and *An*. *squamosus*) than island sites in LB (Table 4), with a mainland incident rate ratio (IRR) estimated at 16.9 (95% CI: (3.5,66.6)) relative to island. Though there was no difference in abundance between mainland and islands at LV, *An*. *rufipes* was only found on island sites, whereas three other anopheline species (*An*. *pharoensis*, *An*. *rhodesiensis*, and *An*. *squamosus*) were specific to the mainland sites. At LB, mainland sites had significantly more catches of *Culex* (15 species) than island sites (χ^2 ^= 4.14, df = 1, *P* = 0.042; Table 2). The mainland IRR was estimated at 7.67 (95% CI: (1.09, 38.7)) relative to island, whilst there was no significant difference between mainland and island in *Culex* catches at LV (χ^2 ^= 0.01, df = 1, *P* = 0.93). Five *Culex* species were specific to the islands (*Cx*. *annulioris*, *Cx*. *sinaiticus*, *Cx*. *terzii*, *Cx*. (*Ncx*.) *adersianus*, and *Cx*. (*Ncx*.) *rima*) whereas three were specific to the mainland towns (*Cx*. *bitaeniorhynchus*, *Cx*. *tenagius*, and *Cx*. *ethiopicus*; Table 2).

*Mansonia* catches were significantly higher at LB than at LV (χ^2 ^= 5.29, df = 1, *P* = 0.02; Table 5). The estimated IRR of *Mansonia* catches for LV relative to LB was 0.14 (95% CI: (0.02,0.7)). In both regions, there were no differences between mainland and island *Mansonia* catches.

Overall, all weather variables per collection month were more consistent at LV than at LB and barometric pressure was markedly lower at LV (Table 7). The wind speeds in both LB and LV were calm (≤0.2 m/s) and light (0.3–1.5 m/s). The islands of both lakes had lower temperatures and higher relative humidity than the mainland sites. In LB, greater numbers of mosquitoes per trap/dip (relative abundance) and mosquito species (richness) were sampled at both island and mainland sites during October, which also had lower relative humidity, and higher temperatures and barometric pressures, than in July. In contrast, LV had higher temperatures and lower wind speeds, barometric pressure, and relative humidity in November than in May, which coincided with higher sampling abundance and lower species richness at island sites, yet lower relative sampling abundance and higher species richness at mainland sites.

**Table 7.** Mean wind speed, temperature, relative humidity, barometric pressure, number of mosquito species (richness), and relative abundance of mosquitoes in the islands and mainland shores of Lake Baringo and Lake Victoria in Kenya

Site	Month (2012)	Wind speed (m/s)	Temp (°C)	Relative humidity (%)	Barometric pressure (hPa)	Species richness	Relative abundance
Lake Baringo							
Island	July	0.01 ± 0.01	22.24 ± 0.52	87.29 ± 2.11	901.09 ± 1.12	3	1.4
	Oct.	1.02 ± 0.22	23.45 ± 0.93	85.28 ± 3.09	973.44 ± 12.35	17	120
Mainland	July	0.2 ± 0.05	24.84 ± 0.68	83.19 ± 1.89	902.07 ± 0.64	12	85
	Oct.	0.08 ± 0.03	26.55 ± 0.37	61.74 ± 1.19	938.9 ± 5.11	25	600.4
Lake Victoria							
Island	May	0.43 ± 0.06	22.7 ± 0.29	81.79 ± 1.19	885.54 ± 0.2	28	27.3
	Nov.	0.07 ± 0.02	26.34 ± 0.21	63.31 ± 0.57	881.54 ± 0.88	26	69.3
Mainland	May	0.41 ± 0.08	23.72 ± 0.36	76.71 ± 0.94	886.63 ± 0.17	22	95.6
	Nov.	0.04 ± 0.02	26.82 ± 0.89	62.52 ± 2.09	884.15 ± 0.22	30	82.3

## Discussion

To identify the diversity of mosquitoes potentially involved in disease transmission cycles in the LV and LB geographic regions of Kenya, we investigated mosquito occurrence, diversity, and distribution in regions with high incidences of malaria and emerging infectious diseases (EIDs) like Rift Valley fever virus. Whereas large numbers of mosquitoes in the LB region are integral to the arboviral outbreaks witnessed in the region, we found greater diversity of mosquito species in the LV region, which is likely to impact on the range of diseases that are likely to occur. Potentially complex transmission cycles involving multiple mosquito species have to be taken into consideration when planning mosquito management strategies.

The mosquito diversity observed in this study (63 species) resulted from sampling at both island (48 species) and mainland (46 species) sites, combining adult and immature stage trapping methods that targeted a broad range of mosquito species. Mosquito populations from islands have been less studied than from mainland towns. As 22% of mosquito species were specific to the islands sampled in our study, exclusion of island mosquito populations can limit surveys. Nonetheless, there could be similar genetic diversity, though likely not relative abundance, among island and mainland mosquito populations. Indeed, Chen et al. (2004) found that populations of *An*. *gambiae* in the Kenyan part of LV were not genetically isolated, irrespective of their origin (mainland or island).

We report 10 of the mosquito species collected for the first time around LB, while 22 have been previously reported in the region (Sang et al. 2010, Omondi et al. 2015). Similarly, out of the 54 mosquito species identified in the LV region, 25 have been previously reported at LV in Kenya (Ochieng et al. 2013, Omondi et al. 2015) and Uganda (Kaddumukasa et al. 2014). Unlike methods employed in our study, human landing collection and pyrethrum spray catches mostly target species that feed exclusively on humans and rest indoors, respectively (Mwangangi et al. 2012, Lutomiah et al. 2013). Also, Linthicum et al. (1985) reported diverse mosquito species that were sampled either only at adult (using light traps and human bait) or immature (using sweep-net and suction of water) stages.

*Mansonia* mosquitoes were the most abundant in both study areas, particularly at LB as found previously (Tchouassi et al. 2012a, Lutomiah et al. 2013). Because *Mansonia* mosquitoes have been implicated as vectors in RVF virus outbreaks in Kenya (Crabtree et al. 2009), their broad distribution across both study areas is of significance to disease transmission and virus outbreak risk. Some of the diseases associated with the *Mansonia* mosquitoes include Bancroftian filariasis (Ughasi et al. 2012), avian malaria (Njabo et al. 2011) and Bunyamwera (Omondi et al. 2015), Ndumu (Ochieng et al. 2013), RVF (Sang et al. 2010), and West Nile (Diallo et al. 2005a) viruses. The high abundance of *Ma*. *africana* and *Ma*. *uniformis* can be attributed to the invasion of aquatic weeds in most Kenyan lakes and to their lakeshore collection points. Ochieng et al. (2013) also reported *Mansonia* species as one of the most abundant mosquitoes collected on the shores of Lakes Naivasha and Victoria. This highlights how changing environmental factors such as increasing presence of aquatic weeds can contribute to disease prevalence (Ofulla et al. 2010).

We identified *Cx*. (*Culex*) *watti* Edwards, *Cx*. (*Neoculex*) *adersianus* Edwards, and *Mi*. (*Etorleptiomyia*) *mediolineata* Theobald from these lake sites for the first time since they were last reported in Kenya in 1985 (Linthicum et al. 1985), 1959 (Teesdale 1959), and 1955 (van Someren et al. 1955). In addition, we captured and identified three mosquito species that have not previously been reported at both lakes, namely, *Culex rima* from Rusinga Island (LV), *Cx*. *perexiguus* from Kampi ya Samaki (LB) and Mfangano Island (LV) using the Dipper, and *Cx*. *sinaiticus* on Mfangano Island (LV) using the CO_2_-baited CDC Lt. Among others, these three species were collected in small numbers (<50 mosquitoes), which may be attributed to mosquito availability in the specific locations and to climatic and ecological factors such as temperature and rainfall (Imbahale et al. 2011) at that time. Previous studies at these lake basins also noted low abundance of some of these species (Lutomiah et al. 2013, Ochieng et al. 2013), and Olanga et al. (2015) attributed low mosquito abundance in Rusinga Island of LV to ongoing malaria interventions. Moreover, we did not find any species of the *Aedeomyia* genus in our LV sites, though they were present in the LB sites and have been reported in different parts of Kenya (van Someren et al. 1955, Sang et al. 2010).

All the *Anopheles gambiae* s.l. analyzed at LB were *An*. *arabiensis*, whereas at LV the majority were *An*. *arabiensis* and a few were *An*. *gambiae* s.s. Meanwhile, the two subspecies are the only ones reported so far around LV (Minakawa et al. 2012), whereas *An*. *arabiensis* has previously been reported as the only one around LB (Mala et al. 2011). Although Omondi et al. (2015) attributed the large numbers of *An*. *arabiensis* to exophily and outdoor placement of the CDC Lt they employed, Olanga et al. (2015) found it to be more abundant both indoors and outdoors compared to *An*. *gambiae* s.s. The *An. rivulorum* collected from Chamaunga Island in LV is one of the two members of the *An*. *funestus* group found around and associated with water hyacinth in LV (Minakawa et al. 2012) and one of the four members found in Kenya (Kamau et al. 2003). *Anopheles rivulorum* rests outside houses and is mainly zoophilic (Wilkes et al. 1996). However, since we did not identify the remaining 237 specimens of the *An*. *funestus* group, we cannot report the exact proportion of the group’s members that were collected in this study.

The quantitative differences in the target stages of the mosquito species may be due to limitations of the different sampling methods. Some species of mosquitoes, such as *Ae. furcifer* and *Ae*. *dentatus*, were captured using the Dipper but not CDC Lt, although the latter was successfully used in other studies to collect these mosquitoes (Tchouassi et al. 2012a). Our dipping method may have been biased toward species whose immature stages forage near the edges of water bodies, especially in large and extensive habitats like lake shores. Similarly, the light from CDC Lt attracts many insects other than mosquitoes at night, which, along with its fan blades, can damage the mosquitoes and make morphological identification difficult (Qiu et al. 2007). The use of CDC Lt to approximate adult mosquito abundance is likely to be biased, even though the color of the light from the standard CDC Lt has been shown to be the most efficient in collecting adult mosquitoes (Tchouassi et al. 2012b). The MM-X trap with Mbita Blend was discontinued because each caught less than five mosquito species per trapping night as compared to the CDC Lt used in this study. Our finding was consistent with that of Olanga et al. (2015) who used the same trap and lure over 432 trap nights yet caught fewer than four mosquitoes per trap per night. In contrary, Nyasembe et al. (2014) reported more mosquito captures by MM-X trap than the CDC Lt, which may be attributed to the counter-flow operation principle and lure release rate of the MM-X trap they used.

Several species of mosquitoes collected have been associated with pathogen transmission. Although some of these were initially regarded as unimportant or of occasional importance, including those of the *An. coustani* complex, *An*. *squamosus*, *Coquillettidia pseudoconopas*, and *Cx. poicilipes* (Gillett 1972), they are currently known as disease vectors either in Kenya (Sang et al. 2010, Mwangangi et al. 2013) or other countries like Cameroon (Njabo et al. 2011), Senegal (Diallo et al. 1999), Mauritania (Diallo et al. 2005b), and Sudan (Seufi and Galal 2010). Some species collected, such as *Aedeomyia furfurea*, *An. rhodesiensis*, and *Cx.* (*Lutzia*) *tigripes*, have been reported as unimportant in disease transmission (Gillett 1972). Pathogen presence in mosquito species does not determine its dissemination capability as seen in *Ma*. *africana* and *Ma*. *uniformis*, which were infected with the West Nile virus (WNV) (Diallo et al. 2005a) but could not transmit the disease (Lutomiah et al. 2011). Although competence studies may be lacking for most mosquito species, vector roles can be inferred based on their abundance and period when sampled (e.g., period of epidemic; Sang et al. 2008). Few species have been tested to competently transmit the pathogens for which they are reported. These include *Cx. quinquefasciatus* Say, *Cx*. *univittatus* Theobald, *Cx*. *vansomereni* Edwards for WNV, *Aedes aegypti* for Bunyamwera virus, *An*. *gambiae* for Bunyamwera and Ngari viruses, and *Cx*. *pipiens*, *Cx*. *antennatus*, and *Cx*. *perexiguus* for RVF (Turell et al. 1996, 2008).

Based on phylogenetic analyses of COI and ITS2 gene sequences, the two *Mansonia* species from LB formed distinct subclusters within those of LV, indicating possible subspeciation of these species to the ecological conditions at LB. Based on COI and ITS2 sequences, *Cx*. *pipiens* separated into two clades, indicating that two subspecies of *Cx*. *pipiens* could exist in the two lake sites. Considering how similar the GenBank COI gene sequences of *Cx*. *pipiens pipiens* and *Cx*. *pipiens quinquefasciatus* are, the distinct *Cx*. *pipiens* clade could represent a different member of the *Cx*. *pipiens* complex or another species of *Culex* that is morphologically similar. However, Cornel et al. (2012) suggested the use of genome-wide single nucleotide polymorphisms to differentiate members of the *Cx*. *pipiens* complex.

*Culex* species can easily be misidentified morphologically (Vesgueiro et al. 2011) by nonexpert taxonomists due to the different forms that can exist within species, such as for *Cx*. *univittatus* (Jupp 1972). Meanwhile, the genetic relationships among *Culex* species is understudied and remain largely unknown (Harbach 2011). Vesgueiro et al. (2011) suggested the use of ITS2 gene sequences to differentiate the *Culex* and *Lutzia* genera, yet sequences from our *Lutzia* specimens cluster among *Culex* sequences. The *Cx*. *perexiguus* that was identified based on the COI sequences of specimens morphologically identified as *Cx*. *univittatus* exists in both study areas, irrespective of mainland or island sites. Jupp (1972) recorded it as an eastern Mediterranean form of *Cx*. *univittatus*. It is a WNV vector and was once known as *Cx*. *univittatus* in Asia (Reuben et al. 1994). Some species can only be differentiated morphologically based on the male genitalia (van Someren 1954). This was the case for *Cx*. *tenagius*, which was identified based on its COI barcode region sequence and is morphologically identical to *Cx*. *quinquefasciatus* (van Someren 1954) and, in this study, to *Cx*. *antennatus*. The species groupings in the recent Cosmopolitan *Culex* classification by Harbach (2011) explained the morphological misidentification faced by mosquito taxonomists, even though it does not include some Kenya mosquitoes. The misidentified mosquitoes in this study were male mosquitoes and mostly *Culex* species, which are usually avoided by taxonomists.

In contrast to the significant COI and ITS2 sequence variation observed within *Mansonia* and *Culex* species, *Ad*. *africana* and *Ad*. *furfurea* had minimal COI gene sequence variation between them, forming a monophyletic cluster on the COI phylogenetic tree. Though not conclusive, this indicates that they may be sibling species that require additional genetic markers for reliable molecular differentiation. Overall, DNA barcoding of mosquito species was able to resolve morphological misidentification of male *Cx*. *watti* and *Aedes dendrophilus keniensis* because the COI sequence divergence between them and their nearest neighbors, *Cx*. *pipiens* and *Ae*. *metallicus*, respectively, as well as between their maximum intraspecific distance and distance to their nearest neighbors was zero (<2%). According to Ashfaq and colleagues (2014), conspecific mosquitoes have a threshold of ≤2.4% sequence divergence, above which they are possible different species. However, COI sequence differences between *Ad*. *africana*, *Ad*. *furfurea*, and among some *Culex* species are below this threshold, despite the fact that they can be morphologically differentiated. Unfortunately, the ITS2 gene of *Ad. furfurea* repeatedly failed to amplify for assessment of its use to molecularly differentiate it from *Ad. africana*.

Weather variables can affect the abundance of different mosquito species. However, despite sampling diverse mosquitoes in different sampling seasons and locations, there was no clear effect of any of the weather variables measured on species richness or relative abundance in this study. Nonetheless, wind speed was calm in both study sites, which has previously been shown to be favorable to sampling higher numbers of mosquitoes and mosquito species (Haddow 1961). We sampled more mosquitoes with greater species richness at LB during the short rains in October, when mosquito average temperatures and barometric pressures were higher and relative humidity was lower than during the long rains in July. Since, Baringo is semiarid, temperatures may be higher when dry, which could be one of the causes of RVF outbreaks in the area (Sang et al. 2010). Chepkorir et al. (2014) demonstrated that high temperature and relative humidity increased the ability of *Ae*. *aegypti* to transmit Dengue-2 virus. It is noteworthy that both Lake environs experienced maximum temperatures above 30°C, yet no outbreak has been recorded around LV, despite the evidence that arboviruses (Sindbis, Ndumu, Usutu, Dengue, West Nile, Yellow fever, and Chikungunya viruses) are circulating (Mease et al. 2011, Ochieng et al. 2013). This is indicative of the complex interactions of factors that contribute to outbreaks.

Since a large proportion of people are fishermen at LV and livestock keepers at LB, outdoor activities predispose them to bites from a number of mosquito species besides *Anopheles*, which transmit malaria. While nonspecific fevers may easily be misdiagnosed for malaria (Kipanga et al. 2014), the risk of exposure to arboviral diseases may be high. As most arboviruses do not have vaccines (Sang and Dunster 2001), vector control is of high importance because mosquito vectors can maintain arboviruses in circulation even after people and animals have been cured of all infections (Joshi et al. 2002). Integrated vector management measures, including those targeting their aquatic stages, can be applied to effectively control vector mosquitoes (Artsob and Lindsay 2008, World Health Organization 2008). However, the ecologies of only very few of these vector species have been studied to date (Sang et al. 2010). This study addresses a critical gap in knowledge of mosquito diversity and contributes data that can be applied to vector control programs, especially in areas of active arbovirus transmission.

In conclusion, differences in species composition and distribution observed at the different sampling sites are likely due to ecological factors. Species diversity of mosquitoes was different on the islands and adjacent mainland shores of LB and LV. The presence of diverse mosquito species identified in this study indicates the richness of mosquito fauna in the areas and the risk of vectored disease transmission to both humans and animals in the two lake regions of Kenya. Three mosquito species (*Cx*. (*Culex*) *sinaiticus*, *Cx*. *perexiguus*, and *Cx*. (*Neoculex*) *rima*) were collected for the first time in Kenya, while significant genetic diversity was observed for *Culex* and *Mansonia* species. For a proper understanding of disease risk, the right tools for species identification are needed. Molecular tools are convenient in correct identification of mosquito species, including cryptic species, though the technique is expensive and labor intensive and thus inappropriate in analysis of large number of samples, especially where pooling is needed. We have demonstrated the value of combining morphological keys with molecular data, in this case the nuclear ITS2 and mitochondrial COI DNA barcoding loci. Further studies sampling on a monthly basis over longer periods may reveal greater mosquito diversity, providing more detail of mosquito ecology in the two Lake regions. Understanding the diversity and abundance of potential mosquito vectors of diseases can provide critical insights to facilitate disease risk forecasting and improve mitigation planning and other management strategies.

## Supplementary Material

Supp. Table 1
