# Supplementary material for: Composition and Genetic Diversity of Mosquitoes (Diptera: Culicidae) on Islands and Mainland Shores of Kenya’s Lakes Victoria and Baringo
Source: J Med Entomol. 2016 Jul 11;53(6):1348–63. doi: 10.1093/jme/tjw102 (PMC5106823; doi:10.1093/jme/tjw102)
Supplement: Supp. Table 1 [file jme_tjw102_index.html]

Supplementary Data | Journal of Medical Entomology

## Supplementary Data

files

- Supplementary Data - zip file
- Supplementary Data - zip file
