# Supplementary material for: Composition and Genetic Diversity of Mosquitoes (Diptera: Culicidae) on Islands and Mainland Shores of Kenya’s Lakes Victoria and Baringo
Source: J Med Entomol. 2016 Jul 11;53(6):1348–63. doi: 10.1093/jme/tjw102 (PMC5106823; doi:10.1093/jme/tjw102)
Supplement: Supp. Table 1 [file suppl_data_01.zip › Supplementary Fig2.docx]

**Supplementary Fig. 2.** Maximum Likelihood phylogenetic tree of the COI gene of the 341 sequenced mosquito species from the Lake Baringo and Lake Victoria study sites. *Cx. perexiguus* and *Cx. tenagius*, revealed by COI gene only, are shaded. Names in black containing ‘voucher’ are sequences included in the analyses from GenBank with their accession numbers. Taxon names in green are from Lake Baringo sites, and those in red are from Lake Victoria sites. The taxa are labeled with name of mosquito species, then GenBank Accession number, with the sequence ID in brackets and the exact site location at the end. Sites ending with ‘Is.’ are Island sites, K. Samaki is Kampi ya Samaki, L. Nyamasare is Luanda Nyamasare.
