# Supplementary material for: Composition and Genetic Diversity of Mosquitoes (Diptera: Culicidae) on Islands and Mainland Shores of Kenya’s Lakes Victoria and Baringo
Source: J Med Entomol. 2016 Jul 11;53(6):1348–63. doi: 10.1093/jme/tjw102 (PMC5106823; doi:10.1093/jme/tjw102)
Supplement: Supp. Table 1 [file suppl_data_01.zip › Supplementary Table 1.docx]

S**upplementary Table 1** The exact location, identity (ID) and GenBank accession numbers of mosquito species on the COI phylogenetic tree

| No. | Morphological ID | Lake site | Exact location | Sample ID on tree | Accession no. |
| --- | --- | --- | --- | --- | --- |
| 1 | *Aedeomyia furfurea* | Lake Baringo | Sirata | M2RM2YA | KU186979 |
| 2 | *Aedeomyia africana* | Lake Baringo | Logumgum | M7YA | KU186980 |
| 3 | *Aedeomyia africana* | Lake Baringo | Logumgum | M8YA | KU186981 |
| 4 | *Aedeomyia africana* | Lake Baringo | Logumgum | M5YA | KU186982 |
| 5 | *Aedeomyia furfurea* | Lake Baringo | Sirata | M4YA | KU186983 |
| 6 | *Aedeomyia furfurea* | Lake Baringo | Sirata | M3YA | KU186984 |
| 7 | *Aedeomyia africana* | Lake Baringo | Logumgum | M6YA | KU186985 |
| 8 | *Aedeomyia furfurea* | Lake Baringo | Sirata | M1YA | KU186986 |
| 9 | *Aedes aegypti* | Lake Victoria | Mbita | M31 | KU186987 |
| 10 | *Aedes aegypti* | Lake Victoria | Mfangano Island | T18 | KU186988 |
| 11 | *Aedes aegypti* | Lake Victoria | Takawiri Island | 1H06 | KU186989 |
| 12 | *Aedes aegypti* | Lake Victoria | Takawiri Island | 91f | KU186990 |
| 13 | *Aedes aegypti* | Lake Victoria | Mfangano Island | T17 | KU186991 |
| 14 | *Aedes hirsutus* | Lake Victoria | Mfangano Island | R8 | KU186992 |
| 15 | *Aedes hirsutus* | Lake Victoria | Chamaunga Island | M92YA | KU186993 |
| 16 | *Aedes hirsutus* | Lake Victoria | Mfangano Island | R9 | KU186994 |
| 17 | *Aedes hirsutus* | Lake Victoria | Rusinga Island | M66YA | KU186995 |
| 18 | *Aedes hirsutus* | Lake Victoria | Ngodhe | M89YA | KU186996 |
| 19 | *Aedes hirsutus* | Lake Victoria | Rusinga Island | M93YA | KU186997 |
| 20 | *Aedes hirsutus* | Lake Victoria | Rusinga Island | M65YA | KU186998 |
| 21 | *Aedes* sp. GPA | Lake Victoria | Mbita | M11YA | KU186999 |
| 22 | *Aedes cumminsi* | Lake Victoria | Rusinga Island | M9YA | KU187000 |
| 23 | *Aedes cumminsi* | Lake Victoria | Chamaunga Island | M10YA | KU187001 |
| 24 | *Aedes* sp. GPA | Lake Victoria | Mbita | LV | KU187002 |
| 25 | *Aedes ochraceus* | Lake Victoria | Chamaunga Island | M18YA | KU187003 |
| 26 | *Aedes vittatus* | Lake Victoria | Mfangano Island | R1 | KU187004 |
| 27 | *Aedes vittatus* | Lake Victoria | Takawiri Island | R17 | KU187005 |
| 28 | *Aedes vittatus* | Lake Victoria | Mfangano Island | R13 | KU187006 |
| 29 | *Aedes vittatus* | Lake Victoria | Takawiri Island | R16 | KU187007 |
| 30 | *Aedes vittatus* | Lake Victoria | Mfangano Island | R2 | KU187008 |
| 31 | *Aedes vittatus* | Lake Victoria | Mfangano Island | R15 | KU187009 |
| 32 | *Aedes vittatus* | Lake Victoria | Mfangano Island | R14 | KU187010 |
| 33 | *Aedes vittatus* | Lake Victoria | Mfangano Island | R4 | KU187011 |
| 34 | *Aedes tarsalis* | Lake Victoria | Sindo | M22YA | KU187012 |
| 35 | *Aedes metallicus* | Lake Victoria | Takawiri Island | R12 | KU187013 |
| 36 | *Aedes metallicus* | Lake Victoria | Mbita | R10 | KU187014 |
| 37 | *Aedes dentatus* | Lake Victoria | Chamaunga Island | M90YA | KU187015 |
| 38 | *Aedes circumluteolus* | Lake Victoria | Mbita | R5 | KU187016 |
| 39 | *Aedes circumluteolus* | Lake Victoria | Mbita | R6 | KU187017 |
| 40 | *Aedes mcintoshi* | Lake Victoria | Chamaunga Island | M17YA | KU187018 |
| 41 | *Culex annulioris* | Lake Victoria | Mfangano Island | R68 | KU187019 |
| 42 | *Culex annulioris* | Lake Victoria | Mfangano Island | R69 | KU187020 |
| 43 | *Culex annulioris* | Lake Victoria | Mfangano Island | R70 | KU187021 |
| 44 | *Culex bitaeniorhynchus* | Lake Baringo | Sirata | M56YA | KU187022 |
| 45 | *Culex* (*Lutzia*) *tigripes* | Lake Victoria | Chamaunga Island | R72 | KU187023 |
| 46 | *Culex* (*Lutzia*) *tigripes* | Lake Victoria | Rusinga Island | T3 | KU187024 |
| 47 | *Culex* (*Lutzia*) *tigripes* | Lake Victoria | Rusinga Island | T4 | KU187025 |
| 48 | *Culex* (*Lutzia*) *tigripes* | Lake Victoria | Chamaunga Island | R71 | KU187026 |
| 49 | *Culex* (*Lutzia*) *tigripes* | Lake Victoria | Rusinga Island | a1 | KU187027 |
| 50 | *Culex* (*Lutzia*) *tigripes* | Lake Baringo | Ruko | 92f | KU187028 |
| 51 | *Culex* (*Lutzia*) *tigripes* | Lake Victoria | Mfangano Island | T2 | KU187029 |
| 52 | *Culex* (*Lutzia*) *tigripes* | Lake Victoria | Mfangano Island | T1 | KU187030 |
| 53 | *Culex* sp. GPE | Lake Baringo | Sirata | 2A04 | KU187031 |
| 54 | *Culex neavei* | Lake Victoria | Mfangano Island | R31 | KU187032 |
| 55 | *Neoculex rima* | Lake Victoria | Rusinga Island | M86YA | KU187033 |
| 56 | *Neoculex rima* | Lake Victoria | Rusinga Island | M85YA | KU187034 |
| 57 | *Neoculex adersianus* | Lake Victoria | Rusinga Island | M81YA | KU187035 |
| 58 | *Neoculex rima* | Lake Victoria | Rusinga Island | M87YA | KU187036 |
| 59 | *Culex antennatus* | Lake Victoria | Mbita | M52YA | KU187037 |
| 60 | *Culex antennatus* | Lake Victoria | Mbita | M54YA | KU187038 |
| 61 | *Culex* sp. GPE | Lake Victoria | Mbita | M70YA | KU187039 |
| 62 | *Culex neavei* | Lake Victoria | Mfangano Island | R29 | KU187040 |
| 63 | *Culex vansomereni* | Lake Victoria | Mbita | R52 | KU187041 |
| 64 | *Culex* sp. GPE | Lake Victoria | Mbita | R33 | KU187042 |
| 65 | *Neoculex adersianus* | Lake Victoria | Rusinga Island | M82YA | KU187043 |
| 66 | *Culex* sp. GPE | Lake Victoria | Mbita | R34 | KU187044 |
| 67 | *Culex* sp. GPE | Lake Baringo | Kampi ya Samaki | M75YA | KU187045 |
| 68 | *Culex neavei* | Lake Victoria | Mfangano Island | R30 | KU187046 |
| 69 | *Culex vansomereni* | Lake Victoria | Mbita | R51 | KU187047 |
| 70 | *Culex antennatus* | Lake Victoria | Mbita | M51YA | KU187048 |
| 71 | *Culex thalassius* | Lake Victoria | Mfangano Island | R44 | KU187049 |
| 72 | *Culex antennatus* | Lake Baringo | Sirata | M48YA | KU187050 |
| 73 | *Neoculex adersianus* | Lake Victoria | Rusinga Island | M83YA | KU187051 |
| 74 | *Culex* sp. GPA | Lake Victoria | Ngodhe Island | T15 | KU187052 |
| 75 | *Culex* sp. GPA | Lake Victoria | Ngodhe Island | T16 | KU187053 |
| 76 | *Culex tenagius* | Lake Baringo | Sirata | M50YA | KU187054 |
| 77 | *Culex* sp. GPB | Lake Victoria | Ngodhe Island | M63YA | KU187055 |
| 78 | *Culex univittatus* | Lake Victoria | Mbita | R49 | KU187056 |
| 79 | *Culex* sp. GPB | Lake Victoria | Mbita | R32 | KU187057 |
| 80 | *Culex* sp. | Lake Baringo | Sirata | M47YA | KU187058 |
| 81 | *Culex univittatus* | Lake Victoria | Mbita | R47 | KU187059 |
| 82 | *Culex univittatus* | Lake Victoria | Mbita | R48 | KU187060 |
| 83 | *Culex simpsoni* | Lake Victoria | Ngodhe Island | 2D11 | KU187061 |
| 84 | *Culex watti* | Lake Victoria | Mfangano Island | 2A05 | KU187062 |
| 85 | *Culex watti* | Lake Victoria | Mbita | M58YA | KU187063 |
| 86 | *Culex watti* | Lake Victoria | Mbita | M59YA | KU187064 |
| 87 | *Culex striatipes* | Lake Victoria | Mfangano Island | R42 | KU187065 |
| 88 | *Culex striatipes* | Lake Victoria | Mfangano Island | R43 | KU187066 |
| 89 | *Culex duttoni* | Lake Victoria | Mfangano Island | R41 | KU187067 |
| 90 | *Culex duttoni* | Lake Victoria | Mfangano Island | R39 | KU187068 |
| 91 | *Culex thalassius* | Lake Victoria | Mfangano Island | R46 | KU187069 |
| 92 | *Culex duttoni* | Lake Victoria | Mfangano Island | R53 | KU187070 |
| 93 | *Culex duttoni* | Lake Victoria | Mfangano Island | R38 | KU187071 |
| 94 | *Culex duttoni* | Lake Victoria | Mfangano Island | R54 | KU187072 |
| 95 | *Culex duttoni* | Lake Baringo | Ruko | 95f1H11 | KU187073 |
| 96 | *Culex duttoni* | Lake Baringo | Ruko | 94f1H10 | KU187074 |
| 97 | *Culex duttoni* | Lake Victoria | Mbita | M57YA | KU187075 |
| 98 | *Culex thalassius* | Lake Victoria | Mfangano Island | R45 | KU187076 |
| 99 | *Culex pipiens* | Lake Victoria | Rusinga Island | 1B10 | KU187077 |
| 100 | *Culex terzii* | Lake Victoria | Rusinga Island | M68YA | KU187078 |
| 101 | *Culex* sp. GPE | Lake Victoria | Mbita | M71YA | KU187079 |
| 102 | *Culex theileri* | Lake Victoria | Mbita | M69YA | KU187080 |
| 103 | *Culex theileri* | Lake Victoria | Rusinga Island | M84YA | KU187081 |
| 104 | *Culex terzii* | Lake Victoria | Rusinga Island | M67YA | KU187082 |
| 105 | *Culex pipiens* | Lake Victoria | Mfangano Island | 1D10 | KU187083 |
| 106 | *Culex* sp. GPD | Lake Victoria | Chamaunga Island | R73 | KU187084 |
| 107 | *Culex zombaensis* | Lake Victoria | Mbita | M72YA | KU187085 |
| 108 | *Culex zombaensis* | Lake Victoria | Mbita | M73YA | KU187086 |
| 109 | *Culex sinaiticus* | Lake Victoria | Ngodhe Island | T26 | KU187087 |
| 110 | *Culex sinaiticus* | Lake Victoria | Mfangano Island | R40 | KU187088 |
| 111 | *Culex nebulosus* | Lake Victoria | Mfangano Island | M78YA | KU187089 |
| 112 | *Culex nebulosus* | Lake Victoria | Mfangano Island | M76YA | KU187090 |
| 113 | *Culex nebulosus* | Lake Victoria | Mfangano Island | M79YA | KU187091 |
| 114 | *Culex nebulosus* | Lake Victoria | Mfangano Island | M77YA | KU187092 |
| 115 | *Mimomyia splendens* | Lake Victoria | Mbita | R66 | KU187093 |
| 116 | *Mimomyia splendens* | Lake Victoria | Mbita | R67 | KU187094 |
| 117 | *Anopheles coustani tenebrosus* | Lake Victoria | Ngodhe | M32YA | KU187095 |
| 118 | *Anopheles coustani tenebrosus* | Lake Victoria | Ngodhe | M33YA | KU187096 |
| 119 | *Anopheles coustani tenebrosus* | Lake Victoria | Rusinga Island | M34YA | KU187097 |
| 120 | *Anopheles coustani ziemanni* | Lake Victoria | Luanda Nyamasare | R28 | KU187098 |
| 121 | *Anopheles coustani tenebrosus* | Lake Victoria | Rusinga Island | M35YA | KU187099 |
| 122 | *Anopheles coustani ziemanni* | Lake Victoria | Luanda Nyamasare | R27 | KU187100 |
| 123 | *Anopheles coustani ziemanni* | Lake Victoria | Luanda Nyamasare | R26 | KU187101 |
| 124 | *Anopheles funestus* | Lake Baringo | Sirata | M23YA | KU187102 |
| 125 | *Anopheles funestus* | Lake Baringo | Sirata | M25YA | KU187103 |
| 126 | *Anopheles funestus* | Lake Victoria | Mbita | M27YA | KU187104 |
| 127 | *Anopheles funestus* | Lake Baringo | Sirata | M91YA | KU187105 |
| 128 | *Anopheles rhodesiensis* | Lake Victoria | Mbita | M28YA | KU187106 |
| 129 | *Anopheles* sp. | Lake Victoria | Mfangano Island | M36YA | KU187107 |
| 130 | *Anopheles gambiae* | Lake Victoria | Mfangano Island | M24YA | KU187108 |
| 131 | *Anopheles gambiae* | Lake Victoria | Mfangano Island | M26YA | KU187109 |
| 132 | *Anopheles squamosus* | Lake Victoria | Ngodhe | M29YA | KU187110 |
| 133 | *Anopheles squamosus* | Lake Victoria | Ngodhe | M30YA | KU187111 |
| 134 | *Coquillettidia metallicus* | Lake Baringo | Sirata | M42YA | KU187112 |
| 135 | *Coquillettidia metallicus* | Lake Baringo | Sirata | M43YA | KU187113 |
| 136 | *Coquillettidia aurites* | Lake Baringo | Sirata | M38YA | KU187114 |
| 137 | *Coquillettidia chrysosoma* | Lake Baringo | Logumgum | M40YA | KU187115 |
| 138 | *Coquillettidia fuscopennata* | Lake Baringo | Nosuguro | M41YA | KU187116 |
| 139 | *Coquillettidia aurites* | Lake Baringo | Sirata | M39YA | KU187117 |
| 140 | *Coquillettidia microannulatus* | Lake Victoria | Chamaunga Island | M44YA | KU187118 |
| 141 | *Coquillettidia versicolor* | Lake Victoria | Ngodhe | M45YA | KU187119 |
| 142 | *Coquillettidia versicolor* | Lake Victoria | Ngodhe | M46YA | KU187120 |
| 143 | *Coquillettidia aurites* | Lake Victoria | Mbita | M37YA | KU187121 |
| 144 | *Mansonia africana* | Lake Baringo | Molo River | M20 | KU187122 |
| 145 | *Mansonia africana* | Lake Baringo | Salabani | M8 | KU187123 |
| 146 | *Mansonia africana* | Lake Baringo | Sirata | M5 | KU187124 |
| 147 | *Mansonia africana* | Lake Baringo | Molo River | M19 | KU187125 |
| 148 | *Mansonia africana* | Lake Baringo | Kokwa Island | M22 | KU187126 |
| 149 | *Mansonia africana* | Lake Baringo | Nosuguro | M26 | KU187127 |
| 150 | *Mansonia africana* | Lake Baringo | Nosuguro | M27 | KU187128 |
| 151 | *Mansonia africana* | Lake Baringo | Kokwa Island | M21 | KU187129 |
| 152 | *Mansonia africana* | Lake Baringo | Sirata | M6 | KU187130 |
| 153 | *Mansonia africana* | Lake Victoria | Mfangano Island | M36 | KU187131 |
| 154 | *Mansonia africana* | Lake Baringo | Logumgum | A9B | KU187132 |
| 155 | *Mansonia africana* | Lake Baringo | Logumgum | M9 | KU187133 |
| 156 | *Mansonia africana* | Lake Baringo | Logumgum | M10 | KU187134 |
| 157 | *Mansonia africana* | Lake Baringo | Logumgum | A6B | KU187135 |
| 158 | *Mansonia africana* | Lake Baringo | Logumgum | A5B | KU187136 |
| 159 | *Mansonia africana* | Lake Baringo | Logumgum | A10B | KU187137 |
| 160 | *Mansonia africana* | Lake Baringo | Ngenyin | A4B | KU187138 |
| 161 | *Mansonia africana* | Lake Baringo | Ngenyin | A1B | KU187139 |
| 162 | *Mansonia africana* | Lake Victoria | Mbita | U4 | KU187140 |
| 163 | *Mansonia africana* | Lake Victoria | Mbita | M2 | KU187141 |
| 164 | *Mansonia africana* | Lake Victoria | Luanda Nyamasare | A4S | KU187142 |
| 165 | *Mansonia africana* | Lake Victoria | Luanda Nyamasare | A6 | KU187143 |
| 166 | *Mansonia africana* | Lake Victoria | Mbita | M4 | KU187144 |
| 167 | *Mansonia africana* | Lake Victoria | Mbita | A3S | KU187145 |
| 168 | *Mansonia africana* | Lake Victoria | Mfangano Island | A7S | KU187146 |
| 169 | *Mansonia africana* | Lake Victoria | Mbita | M32 | KU187147 |
| 170 | *Mansonia africana* | Lake Baringo | Salabani | M7 | KU187148 |
| 171 | *Mansonia africana* | Lake Baringo | Ngenyin | A2B | KU187149 |
| 172 | *Mansonia africana* | Lake Victoria | Mfangano Island | M37 | KU187150 |
| 173 | *Mansonia africana* | Lake Baringo | Logumgum | A8B | KU187151 |
| 174 | *Mansonia africana* | Lake Baringo | Logumgum | A7B | KU187152 |
| 175 | *Mansonia africana* | Lake Victoria | Chamaunga Island | M34 | KU187153 |
| 176 | *Mansonia africana* | Lake Victoria | Luanda Nyamasare | A4 | KU187154 |
| 177 | *Mansonia uniformis* | Lake Baringo | Logumgum | M11 | KU187155 |
| 178 | *Mansonia uniformis* | Lake Baringo | Logumgum | M13 | KU187156 |
| 179 | *Mansonia uniformis* | Lake Baringo | Kampi ya Samaki | M29 | KU187157 |
| 180 | *Mansonia uniformis* | Lake Baringo | Molo River | M17 | KU187158 |
| 181 | *Mansonia uniformis* | Lake Baringo | Kampi ya Samaki | M30 | KU187159 |
| 182 | *Mansonia uniformis* | Lake Baringo | Logumgum | M12 | KU187160 |
| 183 | *Mansonia uniformis* | Lake Baringo | Sirata | M15 | KU187161 |
| 184 | *Mansonia uniformis* | Lake Baringo | Sirata | M16 | KU187162 |
| 185 | *Mansonia uniformis* | Lake Baringo | Kampi ya Samaki | M28 | KU187163 |
| 186 | *Mansonia uniformis* | Lake Baringo | Nosuguro | M23 | KU187164 |
| 187 | *Mansonia uniformis* | Lake Baringo | Sirata | M14 | KU187165 |
| 188 | *Mansonia uniformis* | Lake Baringo | Logumgum | U4B3 | KU187166 |
| 189 | *Mansonia uniformis* | Lake Baringo | Nosuguro | M24 | KU187167 |
| 190 | *Mansonia uniformis* | Lake Baringo | Logumgum | U2B | KU187168 |
| 191 | *Mansonia uniformis* | Lake Baringo | Nosuguro | M25 | KU187169 |
| 192 | *Mansonia uniformis* | Lake Baringo | Logumgum | U10B | KU187170 |
| 193 | *Mansonia uniformis* | Lake Baringo | Logumgum | U6B | KU187171 |
| 194 | *Mansonia uniformis* | Lake Victoria | Mfangano Island | U7S | KU187172 |
| 195 | *Mansonia uniformis* | Lake Victoria | Mfangano Island | U8S | KU187173 |
| 196 | *Mansonia uniformis* | Lake Baringo | Logumgum | U5B | KU187174 |
| 197 | *Mansonia uniformis* | Lake Victoria | Chamaunga Island | uns | KU187175 |
| 198 | *Mansonia uniformis* | Lake Victoria | Mbita | U1S | KU187176 |
| 199 | *Mansonia uniformis* | Lake Victoria | Mfangano Island | U9S | KU187177 |
| 200 | *Mansonia uniformis* | Lake Baringo | Ngenyin | A3B | KU187178 |
| 201 | *Mansonia uniformis* | Lake Baringo | Logumgum | U9B | KU187179 |
| 202 | *Mansonia uniformis* | Lake Baringo | Molo River | M18 | KU187180 |
| 203 | *Mansonia uniformis* | Lake Baringo | Kampi ya Samaki | U1B | KU187181 |
| 204 | *Mansonia uniformis* | Lake Baringo | Logumgum | U7B | KU187182 |
| 205 | *Mansonia uniformis* | Lake Baringo | Logumgum | U8B | KU187183 |
| 206 | *Mansonia uniformis* | Lake Victoria | Chamaunga Island | U6S | KU187184 |
| 207 | *Aedes furcifer* | Lake Victoria | Mbita | M12YA | KU187185 |
| 208 | *Aedes furcifer* | Lake Victoria | Mbita | M13YA | KU187186 |
| 209 | *Aedeomyia africana* | Lake Baringo | Logumgum | 2A10 | KU380398 |
| 210 | *Aedeomyia africana* | Lake Baringo | Logumgum | 2A11 | KU380390 |
| 211 | *Aedeomyia africana* | Lake Baringo | Logumgum | 2A12 | KU380406 |
| 212 | *Aedeomyia africana* | Lake Baringo | Logumgum | 2B01 | KU380456 |
| 213 | *Aedeomyia africana* | Lake Baringo | Logumgum | 2B02 | KU380395 |
| 214 | *Aedes aegypti* | Lake Victoria | Mfangano Island | 1B05 | KU380474 |
| 215 | *Aedes aegypti* | Lake Victoria | Mfangano Island | 1B06 | KU380357 |
| 216 | *Aedes aegypti* | Lake Victoria | Mfangano Island | 1B07 | KU380418 |
| 217 | *Aedes aegypti* | Lake Victoria | Mfangano Island | 1B08 | KU380415 |
| 218 | *Aedes aegypti* | Lake Victoria | Mfangano Island | 1B09 | KU380440 |
| 219 | *Aedes aegypti* | Lake Victoria | Mfangano Island | 1D05 | KU380364 |
| 220 | *Aedes aegypti* | Lake Victoria | Mfangano Island | 1D07 | KU380400 |
| 221 | *Aedes aegypti* | Lake Victoria | Mfangano Island | 1D08 | KU380383 |
| 222 | *Aedes hirsutus* | Lake Victoria | Rusinga Island | 2C12 | KU380443 |
| 223 | *Aedes hirsutus* | Lake Victoria | Rusinga Island | 2D01 | KU380442 |
| 224 | *Aedes hirsutus* | Lake Victoria | Rusinga Island | 2D02 | KU380374 |
| 225 | *Aedes hirsutus* | Lake Victoria | Rusinga Island | 2D03 | KU380380 |
| 226 | *Aedes hirsutus* | Lake Victoria | Rusinga Island | 2E.03 | KU380475 |
| 227 | *Aedes luteocephalus* | Lake Victoria | Mfangano Island | 1C12 | KU380469 |
| 228 | *Aedes luteocephalus* | Lake Victoria | Mfangano Island | 1D01 | KU380427 |
| 229 | *Aedes luteocephalus* | Lake Victoria | Mfangano Island | 1D02 | KU380387 |
| 230 | *Aedes luteocephalus* | Lake Victoria | Mfangano Island | 1D03 | KU380472 |
| 231 | *Aedes luteocephalus* | Lake Victoria | Mfangano Island | 1D04 | KU380354 |
| 232 | *Aedes metallicus* | Lake Victoria | Mbita | 1A06 | KU380376 |
| 233 | *Aedes metallicus* | Lake Victoria | Mbita | 1F04 | KU380467 |
| 234 | *Aedes metallicus* | Lake Victoria | Mbita | 1F05 | KU380353 |
| 235 | *Aedes metallicus* | Lake Victoria | Mbita | 1F06 | KU380347 |
| 236 | *Aedes metallicus* | Lake Victoria | Mbita | 1F07 | KU380373 |
| 237 | *Aedes metallicus* | Lake Victoria | Mbita | 1F08 | KU380438 |
| 238 | *Aedes metallicus* | Lake Victoria | Mfangano Island | 1D09 | KU380468 |
| 239 | *Aedes vittatus* | Lake Victoria | Mfangano Island | 2E.04 | KU380388 |
| 240 | *Aedes vittatus* | Lake Victoria | Mfangano Island | 2E.09 | KU380451 |
| 241 | *Aedes hirsutus* | Lake Victoria | Rusinga Island | 2D06 | KU380385 |
| 242 | *Aedes hirsutus* | Lake Victoria | Rusinga Island | 2D07 | KU380433 |
| 243 | *Anopheles coustani ziemanni* | Lake Baringo | Logumgum | 2C09 | KU380369 |
| 244 | *Anopheles coustani ziemanni* | Lake Baringo | Logumgum | 2C10 | KU380412 |
| 245 | *Anopheles coustani ziemanni* | Lake Baringo | Logumgum | 2C11 | KU380411 |
| 246 | *Anopheles funestus* | Lake Baringo | Logumgum | 2D04 | KU380367 |
| 247 | *Anopheles funestus* | Lake Baringo | Logumgum | 2D05 | KU380404 |
| 248 | *Anopheles gambiae* | Lake Baringo | Kampi ya Samaki | 2A03 | KU380410 |
| 249 | *Anopheles gambiae* | Lake Baringo | Sirata | 2A01 | KU380414 |
| 250 | *Anopheles gambiae* | Lake Baringo | Sirata | 2A02 | KU380403 |
| 251 | *Anopheles gambiae* | Lake Victoria | Mbita | 1D11 | KU380361 |
| 252 | *Anopheles gambiae* | Lake Victoria | Rusinga Island | 1A01 | KU380446 |
| 253 | *Anopheles gambiae* | Lake Victoria | Rusinga Island | 1A02 | KU380466 |
| 254 | *Anopheles gambiae* | Lake Victoria | Rusinga Island | 1A03 | KU380421 |
| 255 | *Anopheles gambiae* | Lake Victoria | Rusinga Island | 1A04 | KU380422 |
| 256 | *Anopheles pharoensis* | Lake Baringo | Sirata | 2C05 | KU380430 |
| 257 | *Anopheles pharoensis* | Lake Baringo | Sirata | 2C07 | KU380435 |
| 258 | *Anopheles pharoensis* | Lake Baringo | Sirata | 2C08 | KU380470 |
| 259 | *Anopheles rufipes* | Lake Victoria | Kibuogi Island | 2B10 | KU380370 |
| 260 | *Culex* sp. | Lake Baringo | Salabani | 2F09 | KU380355 |
| 261 | *Culex annulioris* | Lake Victoria | Mfangano Island | 1C08 | KU380458 |
| 262 | *Culex annulioris* | Lake Victoria | Mfangano Island | 1C09 | KU380464 |
| 263 | *Culex annulioris* | Lake Victoria | Mfangano Island | 1C10 | KU380408 |
| 264 | *Culex annulioris* | Lake Victoria | Mfangano Island | 1C11 | KU380465 |
| 265 | *Culex bitaeniorhynchus* | Lake Baringo | Sirata | 2B09 | KU380407 |
| 266 | *Culex duttoni* | Lake Baringo | Ruko | 1H10 | KU380428 |
| 267 | *Culex duttoni* | Lake Baringo | Ruko | 1H11 | KU380363 |
| 268 | *Culex neavei* | Lake Victoria | Mfangano Island | 2D10 | KU380473 |
| 269 | *Culex* sp. GPA | Lake Victoria | Mbita | 1A07 | KU380455 |
| 270 | *Culex* sp. GPA | Lake Victoria | Mbita | 1A08 | KU380394 |
| 271 | *Culex* sp. GPA | Lake Victoria | Mbita | 1D12 | KU380352 |
| 272 | *Culex pipiens* | Lake Victoria | Rusinga Island | 1B11 | KU380372 |
| 273 | *Culex pipiens* | Lake Victoria | Rusinga Island | 1B12 | KU380444 |
| 274 | *Culex pipiens* | Lake Victoria | Rusinga Island | 1C01 | KU380366 |
| 275 | *Culex pipiens* | Lake Victoria | Rusinga Island | 1C02 | KU380431 |
| 276 | *Culex* sp. GPA | Lake Victoria | Rusinga Island | 1C04 | KU380413 |
| 277 | *Culex* sp. GPA | Lake Victoria | Rusinga Island | 1C05 | KU380377 |
| 278 | *Culex pipiens* | Lake Victoria | Rusinga Island | 1E.09 | KU380349 |
| 279 | *Culex pipiens* | Lake Victoria | Rusinga Island | 1E.10 | KU380375 |
| 280 | *Culex pipiens* | Lake Victoria | Rusinga Island | 1E.11 | KU380401 |
| 281 | *Culex pipiens* | Lake Victoria | Rusinga Island | 1F01 | KU380389 |
| 282 | *Culex pipiens* | Lake Victoria | Ungoye | 1E.02 | KU380426 |
| 283 | *Culex pipiens* | Lake Victoria | Ungoye | 1E.03 | KU380396 |
| 284 | *Culex pipiens* | Lake Victoria | Ungoye | 1E.04 | KU380416 |
| 285 | *Culex pipiens* | Lake Victoria | Ungoye | 1E.05 | KU380381 |
| 286 | *Culex pipiens* | Lake Victoria | Ungoye | 1E.08 | KU380362 |
| 287 | *Aedes hirsutus* | Lake Victoria | Chamaunga Island | 2B11 | KU380459 |
| 288 | *Aedes hirsutus* | Lake Victoria | Chamaunga Island | 2B12 | KU380386 |
| 289 | *Aedes hirsutus* | Lake Victoria | Chamaunga Island | 2C01 | KU380384 |
| 290 | *Aedes hirsutus* | Lake Victoria | Chamaunga Island | 2C02 | KU380419 |
| 291 | *Culex* sp. GPB | Lake Victoria | Ngodhe Island | 2E.01 | KU380463 |
| 292 | *Culex* sp. GPC | Lake Victoria | Ungoye | 2D12 | KU380436 |
| 293 | *Culex striatipes* | Lake Victoria | Mbita | 2C03 | KU380424 |
| 294 | *Culex striatipes* | Lake Victoria | Mbita | 2C04 | KU380397 |
| 295 | *Culex perexiguus* | Lake Baringo | Kampi ya Samaki | 2A06 | KU380423 |
| 296 | *Culex perexiguus* | Lake Baringo | Kampi ya Samaki | 2A07 | KU380348 |
| 297 | *Culex perexiguus* | Lake Baringo | Kampi ya Samaki | 2A08 | KU380476 |
| 298 | *Culex perexiguus* | Lake Baringo | Kampi ya Samaki | 2A09 | KU380382 |
| 299 | *Culex univittatus* | Lake Victoria | Mbita | 1F02 | KU380425 |
| 300 | *Culex perexiguus* | Lake Victoria | Mfangano Island | 1A05 | KU380445 |
| 301 | *Culex sinaiticus* | Lake Victoria | Mfangano Island | 1E.06 | KU380450 |
| 302 | *Culex sinaiticus* | Lake Victoria | Mfangano Island | 1E.07 | KU380392 |
| 303 | *Culex vansomereni* | Lake Victoria | Mfangano Island | 2B03 | KU380350 |
| 304 | *Culex pipiens* | Lake Victoria | Ungoye | 1E.01 | KU380437 |
| 305 | *Culex* (*Lutzia*) *tigripes* | Lake Baringo | Ruko | 1H08 | KU380452 |
| 306 | *Culex* (*Lutzia*) *tigripes* | Lake Baringo | Ruko | 1H09 | KU380439 |
| 307 | *Culex* (*Lutzia*) *tigripes* | Lake Baringo | Ruko | 2B04 | KU380351 |
| 308 | *Culex* (*Lutzia*) *tigripes* | Lake Victoria | Mbita | 1F03 | KU380477 |
| 309 | *Mansonia africana* | Lake Baringo | Kampi ya Samaki | 1G09 | KU380405 |
| 310 | *Mansonia africana* | Lake Baringo | Kampi ya Samaki | 1G10 | KU380402 |
| 311 | *Mansonia africana* | Lake Baringo | Kampi ya Samaki | 1G12 | KU380448 |
| 312 | *Mansonia africana* | Lake Baringo | Logumgum | 1H01 | KU380479 |
| 313 | *Mansonia africana* | Lake Baringo | Logumgum | 1H02 | KU380432 |
| 314 | *Mansonia africana* | Lake Baringo | Logumgum | 1H03 | KU380417 |
| 315 | *Mansonia africana* | Lake Baringo | Logumgum | 1H04 | KU380379 |
| 316 | *Mansonia africana* | Lake Baringo | Logumgum | 1H05 | KU380371 |
| 317 | *Mansonia africana* | Lake Victoria | Lwanda Nyamasare | 1A10 | KU380393 |
| 318 | *Mansonia africana* | Lake Victoria | Lwanda Nyamasare | 1A11 | KU380429 |
| 319 | *Mansonia africana* | Lake Victoria | Mbita | 1G07 | KU380478 |
| 320 | *Mansonia africana* | Lake Victoria | Mbita | 1C06 | KU380365 |
| 321 | *Mansonia uniformis* | Lake Baringo | Kampi ya Samaki | 1G11 | KU380360 |
| 322 | *Mansonia uniformis* | Lake Baringo | Kampi ya Samaki | 1F09 | KU380399 |
| 323 | *Mansonia uniformis* | Lake Baringo | Logumgum | 1F10 | KU380358 |
| 324 | *Mansonia uniformis* | Lake Baringo | Logumgum | 1F11 | KU380409 |
| 325 | *Mansonia uniformis* | Lake Baringo | Logumgum | 1F12 | KU380449 |
| 326 | *Mansonia uniformis* | Lake Baringo | Logumgum | 1G01 | KU380420 |
| 327 | *Mansonia uniformis* | Lake Baringo | Logumgum | 1G02 | KU380368 |
| 328 | *Mansonia uniformis* | Lake Baringo | Logumgum | 1G03 | KU380461 |
| 329 | *Mansonia uniformis* | Lake Baringo | Logumgum | 1G04 | KU380356 |
| 330 | *Mansonia uniformis* | Lake Baringo | Logumgum | 1G05 | KU380378 |
| 331 | *Mansonia uniformis* | Lake Baringo | Logumgum | 1G06 | KU380441 |
| 332 | *Mansonia uniformis* | Lake Victoria | Lwanda Nyamasare | 1A09 | KU380460 |
| 333 | *Mimomyia splendens* | Lake Baringo | Kampi ya Samaki | 2B05 | KU380454 |
| 334 | *Mimomyia splendens* | Lake Baringo | Kampi ya Samaki | 2B06 | KU380359 |
| 335 | *Mimomyia splendens* | Lake Baringo | Lempakany Island | 1C03 | KU380391 |
| 336 | *Mimomyia splendens* | Lake Baringo | Sirata | 2B07 | KU380434 |
| 337 | *Mimomyia splendens* | Lake Baringo | Sirata | 2B08 | KU380447 |
| 338 | *Culex* (*Neoculex*) *adersianus* | Lake Victoria | Rusinga Island | 1B02 | KU380453 |
| 339 | *Culex* (*Neoculex*) *adersianus* | Lake Victoria | Rusinga Island | 1B03 | KU380457 |
| 340 | *Culex* (*Neoculex*) *rima* | Lake Victoria | Rusinga Island | 1A12 | KU380462 |
| 341 | *Culex* (*Neoculex*) *rima* | Lake Victoria | Rusinga Island | 1B01 | KU380471 |

Numbers 209 to 341 are samples with corresponding photographs on BOLD in the DS-KMOSQBV dataset
