# Supplementary material for: Composition and Genetic Diversity of Mosquitoes (Diptera: Culicidae) on Islands and Mainland Shores of Kenya’s Lakes Victoria and Baringo
Source: J Med Entomol. 2016 Jul 11;53(6):1348–63. doi: 10.1093/jme/tjw102 (PMC5106823; doi:10.1093/jme/tjw102)
Supplement: Supp. Table 1 [file suppl_data_01.zip › Supplementary Table 2.docx]

**Supplementary Table 2** Mosquito species in this study that have been reported as disease vectors

| **Pathogen** | **Disease pathogen** | **References** |
| --- | --- | --- |
| Helminth | Dog heartworm | *Cx*. *theileri* (Santa-Ana et al. 2006) |
|  | Filarial worm | *Ae*. *aegypti* (Gillett 1972), *An*. *funestus* (Gillett 1972, World Health Organization 2013), *An*. *gambiae* s.l. (Gillett 1972, World Health Organization 2013), *Cx*. *antennatus* (World Health Organization 2013), *Cx*. *bitaeniorhynchus*^§^ (World Health Organization 2013), *Cx*. *pipiens* (Gillett 1972, World Health Organization 2013), *Ma*. *africana* (Gillett 1972, Ughasi et al. 2012), *Ma*. *uniformis* (Gillett 1972, Ughasi et al. 2012, World Health Organization 2013) |
| Protozoan *Plasmodium* | Avian malaria parasite | *Ae*. *mcintoshi* (Njabo et al. 2011), *Cq*. *aurites* (Njabo et al. 2011), *Cq*. *metallicus* (Njabo et al. 2011), *Cq*. *pseudoconopas* (Njabo et al. 2011), *Cx*. *annulioris* (Njabo et al. 2011), *Cx*. *neavei* (Njabo et al. 2011), *Cx*. *poicilipes* (Njabo et al. 2011), *Cx*. *vansomereni* (Njabo et al. 2011), *Ma*. *uniformis* (Njabo et al. 2011) |
|  | Human malaria parasite | ***An*. *coustani*** (Gillies and De Meillon 1968, Mwangangi et al. 2013), *An*. *tenebrosus* (Aranda et al. 2005), *An*. *ziemanni* (Gillies and De Meillon 1968), ***An*. *arabiensis●*** (Gillies and Coetzee 1987, Mwangangi et al. 2013, Olanga et al. 2015), *An*. *gambiae* s.s. (Gillies and Coetzee 1987), ***An*. *funestus●*** (Gillies and De Meillon 1968, Gillett 1972, Gillies and Coetzee 1987, Mwangangi et al. 2013, Olanga et al. 2015), *An*. *rivulorum* (Wilkes et al. 1996), *An*. *pharoensis* (Gillies and De Meillon 1968, Gillett 1972, Gillies and Coetzee 1987), *An*. *squamosus* (Gillies and De Meillon 1968, Gillies and Coetzee 1987), *An*. *rufipes* (Gillies and De Meillon 1968) |
| Virus | Babanki virus | ***Ae*. *circumluteolus*** (Crabtree et al. 2009), *Ae*. *mcintoshi* (Crabtree et al. 2009, Ochieng et al. 2013), ***Ae*. *ochraceus*** (Ochieng et al. 2013), *Cx*. *univittatus* group (Gordon et al. 1992), ***Cx*. *vansomereni*** (Ochieng et al. 2013), ***Cx*. *zombaensis*** (Ochieng et al. 2013), *Mi*. *hispida* (Traore-Lamizana et al. 1994) |
|  | Bagaza virus | *Cx*. *neavei* (Traore-Lamizana et al. 1994, Diallo et al. 2005a), *Cx*. *poicilipes* (Traore-Lamizana et al. 1994, Diallo et al. 2005a), *Cx*. *univittatus* group (Gordon et al. 1992), *Mi*. *hispida* (Traore-Lamizana et al. 1994), *Mi*. *splendens* (Traore-Lamizana et al. 1994) |
|  | Bangui virus | *An*. *pharoensis* (Gordon et al. 1992) |
|  | Bunyamwera virus | ***Ae*. *aegypti*** (Odhiambo et al. 2014), *Ae*. *circumluteolus* (Gillett 1972, Jupp 1996), ***Ae*. *mcintoshi*** (Crabtree et al. 2009, Ochieng et al. 2013), ***Ae*. *ochraceus*** (Crabtree et al. 2009), ***An*. *funestus*** (Ochieng et al. 2013), ***An*. *gambiae*** (Odhiambo et al. 2014), ***Ma*. *africana*●** (Gillett 1972, Omondi et al. 2015), ***Ad*. *africana*●** (Omondi et al. 2015), ***An*. *coustani*●** (Omondi et al. 2015) |
|  | Bwamba virus | *An*. *funestus* (Gillett 1972, Lutwama et al. 1999), *Ma*. *africana* (Gillett 1972), *Ma*. *uniformis* (Gillett 1972) |
|  | Chikungunya virus | *Ae*. *aegypti* (Gillett 1972, Sang et al. 2008), *Ae*. *furcifer* (Jupp 1996, Diallo et al. 1999), *Ae*. *luteocephalus* (Diallo et al. 1999), *Ae*. *vittatus* (Diallo et al. 1999), *An*. *coustani* (Diallo et al. 1999), *An*. *rufipes* (Diallo et al. 1999), *Cx*. *ethiopicus* (Diallo et al. 1999), *Cx*. *pipiens* (Gillett 1972), *Cq*. *fuscopennata* (Gillett 1972), *Ma*. *africana* (Gillett 1972), *Ma*. *uniformis* (Gillett 1972) |
|  | Dengue virus | *Ae*. *aegypti* (Gillett 1972, Joshi et al. 2002, Chepkorir et al. 2014), *Ae*. *furcifer* (Diallo et al. 2005b), *Ae*. *luteocephalus* (Diallo et al. 2005b) |
|  | Kamese virus | *Ae*. *circumluteolus* (Gillett 1972), *Cx*. *annulioris* (Gillett 1972) |
|  | Lunyo virus | *Ae*. *aegypti* (Gillett 1972) |
|  | Middleburg virus | *Ae*. *circumluteolus* (Gillett 1972) |
|  | Ndumu virus | ***Ae*. *circumluteolus*** (Gillett 1972, Crabtree et al. 2009), ***Ae*. *mcintoshi*** (Crabtree et al. 2009, Ochieng et al. 2013), ***Ae*. *ochraceus*** (Crabtree et al. 2009, Ochieng et al. 2013), ***Ma*. *africana●*** (Ochieng et al. 2013), ***Ma*. *uniformis●*** (Gillett 1972, Crabtree et al. 2009) |
|  | Ngari virus | *Ae*. *hirsutus* (Gordon et al. 1992), ***Ae*. *mcintoshi*** (Ochieng et al. 2013), ***An*. *funestus*** (Ochieng et al. 2013), ***An*. *gambiae* complex** (Gordon et al. 1992, Odhiambo et al. 2014), *An*. *pharoensis* (Gordon et al. 1992), *Cx*. *antennatus* (Gordon et al. 1992), *Cx*. *poicilipes* (Gordon et al. 1992) |
|  | Ntaya virus | *Cx*. *annulioris* (Gillett 1972) |
|  | Nyando virus | *An*. *funestus* (Gillett 1972, Lutwama et al. 1999) |
|  | O’nyong-nyong virus | *An*. *funestus* (Gillies and De Meillon 1968, Gillett 1972, Lutwama et al. 1999), *An*. *gambiae* (Gillies and De Meillon 1968, Gillett 1972), *Ma*. *uniformis* (Lutwama et al. 1999) |
|  | Pongola virus | ***Ae*. *circumluteolus*** (Gillett 1972, Jupp 1996, Crabtree et al. 2009), ***Ae*. *mcintoshi*** (Crabtree et al. 2009, Ochieng et al. 2013), *Ma*. *africana* (Gillett 1972) |
|  | Rift Valley Fever virus | *Ae*. *aegypti* (Seufi and Galal 2010), ***Ae*. *circumluteolus*** (Gillett 1972, Turell et al. 2008, Sang et al. 2010), ***Ae*. *cumminsi*** (Linthicum et al. 1985), ***Ae*. *mcintoshi*** (Linthicum et al. 1985, Turell et al. 2008, Sang et al. 2010, Tchouassi et al. 2012b), ***Ae*. *ochraceus*** (Sang et al. 2010, Tchouassi et al. 2012b), *An*. *coustani* (Seufi and Galal 2010), *An*. *arabiensis* (Seufi and Galal 2010), ***An*. *pharoensis*** (Linthicum et al. 1985), ***An*. *squamosus*** (Sang et al. 2010), ***Cx*. *antennatus*** (Linthicum et al. 1985, Turell et al. 1996, Turell et al. 2008), ***Cx*. *bitaeniorhynchus*** (Sang et al. 2010), *Cx*. *perexiguus* (Turell et al. 1996), ***Cx*. *pipiens* complex**● (Turell et al. 1996, Turell et al. 2008, Seufi and Galal 2010), ***Cx*. *poicilipes*** (Diallo et al. 2005a, Sang et al. 2010, Seufi and Galal 2010), *Cx*. *theileri* (Jupp 1996), ***Cx*. *univittatus●*** (Sang et al. 2010), ***Cx*. *vansomereni*** (Linthicum et al. 1985), ***Cx*. *zombaensis*** (Linthicum et al. 1985, Logan et al. 1991, Jupp 1996), *Cq*. *fuscopennata* (Daubney and Hudson 1933, Gillett 1972), *Cq*. *microannulata* (Daubney and Hudson 1933), *Cq*. *versicolor* (Daubney and Hudson 1933), ***Ma*. *africana●*** (Gillett 1972, Logan et al. 1991, Sang et al. 2010), ***Ma*. *uniformis●*** (Gillett 1972, Sang et al. 2010) |
|  | Sanar virus | *Cx*. *poicilipes* (Diallo et al. 2005a) |
|  | Semliki Forest virus | ***Ae*. *circumluteolus*** (Crabtree et al. 2009), ***Ae*. *mcintoshi*** (Crabtree et al. 2009), ***Ae*. *ochraceus*** (Crabtree et al. 2009) |
|  | Simbu virus | *Ae*. *circumluteolus* (Gillett 1972) |
|  | Sindbis Virus | *An*. *pharoensis* (Gillett 1972), ***Cx*. *pipiens* complex●** (Crabtree et al. 2009, Omondi et al. 2015), *Cx*. *theileri* (Jupp 1985), *Cx*. *univittatus* (Jupp 1996), ***Cq*. *fuscopennata●*** (Gillett 1972, Ochieng et al. 2013), *Ma*. *africana* (Gillett 1972) |
|  | Spondweni virus | *Ae*. *circumluteolus* (Gillett 1972, Jupp 1996), *Ae*. *cumminsi* (Gillett 1972), *Ma*. *africana* (Gillett 1972), *Ma*. *uniformis* (Gillett 1972) |
|  | Tanga virus | *An*. *funestus* (Gillett 1972) |
|  | Uganda S virus | *Ae*. *aegypti* (Gillett 1972) |
|  | Usutu virus | ***Cx*. *pipiens●*** (Ochieng et al. 2013), *Cq*. *aurites* (Haddow et al. 1964, Gillett 1972) |
|  | Wesselsbron virus | *Ae*. *circumluteolus* (Gillett 1972), *Ma*. *uniformis* (Gillett 1972) |
|  | West Nile Virus | *Ad*. *africana* (Traore-Lamizana et al. 1994), *Cx*. *neavei* (Traore-Lamizana et al. 1994), ***Cx*. *quinquefasciatus*** (Gillett 1972, Lutomiah et al. 2011), *Cx*. *poicilipes* (Traore-Lamizana et al. 1994), ***Cx*. *univittatus*** (Jupp 1996, Lutomiah et al. 2011, Ochieng et al. 2013), ***Cx*. *vansomereni*** (Lutomiah et al. 2011), *Cq*. *metallicus* (Gillett 1972), *Ma*. *uniformis* (Diallo et al. 2005a), *Mi*. *hispida* (Traore-Lamizana et al. 1994), *Mi*. *splendens* (Traore-Lamizana et al. 1994), |
|  | Yellow Fever virus | *Ae*. *aegypti* (Gillett 1972, World Health Organization 2014), *Ae*. *dentatus* (World Health Organization 2014), *Ae*. *furcifer* (World Health Organization 2014), *Ae*. *luteocephalus* (World Health Organization 2014), *Ae*. *metallicus* (Gillett 1972, World Health Organization 2014), *Ae*. *simpsoni* (Gillett 1972, World Health Organization 2014), *Ae*. *tarsalis* (World Health Organization 2014), *Ae*. *vittatus* (Gillett 1972, World Health Organization 2014), *Cx*. *pipiens* (World Health Organization 2014), *Cx*. *thalassius* (World Health Organization 2014), *Cq*. *fuscopennata* (World Health Organization 2014), *Ma*. *africana* (Gillett 1972, World Health Organization 2014) |
|  | Zika virus | *Ae. africanus* (Haddow et al. 1964) |

Names in bold are mosquito species from which pathogen(s) have been isolated in Kenya.

^§^ “Doubtfully or rarely implicated in transmission” (World Health Organization 2013). ●Reported pathogen(s) has been isolated from these mosquitoes in Kenya’s Lake Baringo or Lake Victoria environs
