# Supplementary material for: Composition and Genetic Diversity of Mosquitoes (Diptera: Culicidae) on Islands and Mainland Shores of Kenya’s Lakes Victoria and Baringo
Source: J Med Entomol. 2016 Jul 11;53(6):1348–63. doi: 10.1093/jme/tjw102 (PMC5106823; doi:10.1093/jme/tjw102)
Supplement: Supp. Table 1 [file suppl_data_02.zip › Revised Supplementary Table 3.docx]

**Supplementary Table 3** Mosquito species in this study that have been reported as disease vectors

| **Pathogen** | **Disease pathogen** | **References** |
| --- | --- | --- |
| Helminth | Dog heartworm | *Cx*. *theileri* (Santa-Ana et al. 2006) |
|  | Filarial worm | *Ae*. *aegypti* (Gillett 1972), *An*. *funestus* (Gillett 1972, World Health Organization 2013), *An*. *gambiae* s.l. (Gillett 1972, World Health Organization 2013), *Cx*. *antennatus* (World Health Organization 2013), *Cx*. *bitaeniorhynchus*^§^ (World Health Organization 2013), *Cx*. *pipiens* (Gillett 1972, World Health Organization 2013), *Ma*. *africana* (Gillett 1972, Ughasi et al. 2012), *Ma*. *uniformis* (Gillett 1972, Ughasi et al. 2012, World Health Organization 2013) |
| Protozoan *Plasmodium* | Avian malaria parasite | *Ae*. *mcintoshi* (Njabo et al. 2011), *Cq*. *aurites* (Njabo et al. 2011), *Cq*. *metallicus* (Njabo et al. 2011), *Cq*. *pseudoconopas* (Njabo et al. 2011), *Cx*. *annulioris* (Njabo et al. 2011), *Cx*. *neavei* (Njabo et al. 2011), *Cx*. *poicilipes* (Njabo et al. 2011), *Cx*. *vansomereni* (Njabo et al. 2011), *Ma*. *uniformis* (Njabo et al. 2011) |
|  | Human malaria parasite | ***An*. *coustani*** (Gillies and De Meillon 1968, Mwangangi et al. 2013), *An*. *tenebrosus* (Aranda et al. 2005), *An*. *ziemanni* (Gillies and De Meillon 1968), ***An*. *arabiensis●*** (Gillies and Coetzee 1987, Mwangangi et al. 2013, Olanga et al. 2015), *An*. *gambiae* s.s. (Gillies and Coetzee 1987), ***An*. *funestus●*** (Gillies and De Meillon 1968, Gillett 1972, Gillies and Coetzee 1987, Mwangangi et al. 2013, Olanga et al. 2015), *An*. *rivulorum* (Wilkes et al. 1996), *An*. *pharoensis* (Gillies and De Meillon 1968, Gillett 1972, Gillies and Coetzee 1987), *An*. *squamosus* (Gillies and De Meillon 1968, Gillies and Coetzee 1987), *An*. *rufipes* (Gillies and De Meillon 1968) |
| Virus | Babanki virus | ***Ae*. *circumluteolus*** (Crabtree et al. 2009), *Ae*. *mcintoshi* (Crabtree et al. 2009, Ochieng et al. 2013), ***Ae*. *ochraceus*** (Ochieng et al. 2013), *Cx*. *univittatus* group (Gordon et al. 1992), ***Cx*. *vansomereni*** (Ochieng et al. 2013), ***Cx*. *zombaensis*** (Ochieng et al. 2013), *Mi*. *hispida* (Traore-Lamizana et al. 1994) |
|  | Bagaza virus | *Cx*. *neavei* (Traore-Lamizana et al. 1994, Diallo et al. 2005a), *Cx*. *poicilipes* (Traore-Lamizana et al. 1994, Diallo et al. 2005a), *Cx*. *univittatus* group (Gordon et al. 1992), *Mi*. *hispida* (Traore-Lamizana et al. 1994), *Mi*. *splendens* (Traore-Lamizana et al. 1994) |
|  | Bangui virus | *An*. *pharoensis* (Gordon et al. 1992) |
|  | Bunyamwera virus | ***Ae*. *aegypti*** (Odhiambo et al. 2014), *Ae*. *circumluteolus* (Gillett 1972, Jupp 1996), ***Ae*. *mcintoshi*** (Crabtree et al. 2009, Ochieng et al. 2013), ***Ae*. *ochraceus*** (Crabtree et al. 2009), ***An*. *funestus*** (Ochieng et al. 2013), ***An*. *gambiae*** (Odhiambo et al. 2014), ***Ma*. *africana*●** (Gillett 1972, Omondi et al. 2015), ***Ad*. *africana*●** (Omondi et al. 2015), ***An*. *coustani*●** (Omondi et al. 2015) |
|  | Bwamba virus | *An*. *funestus* (Gillett 1972, Lutwama et al. 1999), *Ma*. *africana* (Gillett 1972), *Ma*. *uniformis* (Gillett 1972) |
|  | Chikungunya virus | *Ae*. *aegypti* (Gillett 1972, Sang et al. 2008), *Ae*. *furcifer* (Jupp 1996, Diallo et al. 1999), *Ae*. *luteocephalus* (Diallo et al. 1999), *Ae*. *vittatus* (Diallo et al. 1999), *An*. *coustani* (Diallo et al. 1999), *An*. *rufipes* (Diallo et al. 1999), *Cx*. *ethiopicus* (Diallo et al. 1999), *Cx*. *pipiens* (Gillett 1972), *Cq*. *fuscopennata* (Gillett 1972), *Ma*. *africana* (Gillett 1972), *Ma*. *uniformis* (Gillett 1972) |
|  | Dengue virus | *Ae*. *aegypti* (Gillett 1972, Joshi et al. 2002, Chepkorir et al. 2014), *Ae*. *furcifer* (Diallo et al. 2005b), *Ae*. *luteocephalus* (Diallo et al. 2005b) |
|  | Kamese virus | *Ae*. *circumluteolus* (Gillett 1972), *Cx*. *annulioris* (Gillett 1972) |
|  | Lunyo virus | *Ae*. *aegypti* (Gillett 1972) |
|  | Middleburg virus | *Ae*. *circumluteolus* (Gillett 1972) |
|  | Ndumu virus | ***Ae*. *circumluteolus*** (Gillett 1972, Crabtree et al. 2009), ***Ae*. *mcintoshi*** (Crabtree et al. 2009, Ochieng et al. 2013), ***Ae*. *ochraceus*** (Crabtree et al. 2009, Ochieng et al. 2013), ***Ma*. *africana●*** (Ochieng et al. 2013), ***Ma*. *uniformis●*** (Gillett 1972, Crabtree et al. 2009) |
|  | Ngari virus | *Ae*. *hirsutus* (Gordon et al. 1992), ***Ae*. *mcintoshi*** (Ochieng et al. 2013), ***An*. *funestus*** (Ochieng et al. 2013), ***An*. *gambiae* complex** (Gordon et al. 1992, Odhiambo et al. 2014), *An*. *pharoensis* (Gordon et al. 1992), *Cx*. *antennatus* (Gordon et al. 1992), *Cx*. *poicilipes* (Gordon et al. 1992) |
|  | Ntaya virus | *Cx*. *annulioris* (Gillett 1972) |
|  | Nyando virus | *An*. *funestus* (Gillett 1972, Lutwama et al. 1999) |
|  | O’nyong-nyong virus | *An*. *funestus* (Gillies and De Meillon 1968, Gillett 1972, Lutwama et al. 1999), *An*. *gambiae* (Gillies and De Meillon 1968, Gillett 1972), *Ma*. *uniformis* (Lutwama et al. 1999) |
|  | Pongola virus | ***Ae*. *circumluteolus*** (Gillett 1972, Jupp 1996, Crabtree et al. 2009), ***Ae*. *mcintoshi*** (Crabtree et al. 2009, Ochieng et al. 2013), *Ma*. *africana* (Gillett 1972) |
|  | Rift Valley fever virus | *Ae*. *aegypti* (Seufi and Galal 2010), ***Ae*. *circumluteolus*** (Gillett 1972, Turell et al. 2008, Sang et al. 2010), ***Ae*. *cumminsi*** (Linthicum et al. 1985), ***Ae*. *mcintoshi*** (Linthicum et al. 1985, Turell et al. 2008, Sang et al. 2010, Tchouassi et al. 2012), ***Ae*. *ochraceus*** (Sang et al. 2010, Tchouassi et al. 2012), *An*. *coustani* (Seufi and Galal 2010), *An*. *arabiensis* (Seufi and Galal 2010), ***An*. *pharoensis*** (Linthicum et al. 1985), ***An*. *squamosus*** (Sang et al. 2010), ***Cx*. *antennatus*** (Linthicum et al. 1985, Turell et al. 1996, Turell et al. 2008), ***Cx*. *bitaeniorhynchus*** (Sang et al. 2010), *Cx*. *perexiguus* (Turell et al. 1996), ***Cx*. *pipiens* complex**● (Turell et al. 1996, Turell et al. 2008, Seufi and Galal 2010), ***Cx*. *poicilipes*** (Diallo et al. 2005a, Sang et al. 2010, Seufi and Galal 2010), *Cx*. *theileri* (Jupp 1996), ***Cx*. *univittatus●*** (Sang et al. 2010), ***Cx*. *vansomereni*** (Linthicum et al. 1985), ***Cx*. *zombaensis*** (Linthicum et al. 1985, Logan et al. 1991, Jupp 1996), *Cq*. *fuscopennata* (Daubney and Hudson 1933, Gillett 1972), *Cq*. *microannulata* (Daubney and Hudson 1933), *Cq*. *versicolor* (Daubney and Hudson 1933), ***Ma*. *africana●*** (Gillett 1972, Logan et al. 1991, Sang et al. 2010), ***Ma*. *uniformis●*** (Gillett 1972, Sang et al. 2010) |
|  | Sanar virus | *Cx*. *poicilipes* (Diallo et al. 2005a) |
|  | Semliki Forest virus | ***Ae*. *circumluteolus*** (Crabtree et al. 2009), ***Ae*. *mcintoshi*** (Crabtree et al. 2009), ***Ae*. *ochraceus*** (Crabtree et al. 2009) |
|  | Simbu virus | *Ae*. *circumluteolus* (Gillett 1972) |
|  | Sindbis virus | *An*. *pharoensis* (Gillett 1972), ***Cx*. *pipiens* complex●** (Crabtree et al. 2009, Omondi et al. 2015), *Cx*. *theileri* (Jupp 1985), *Cx*. *univittatus* (Jupp 1996), ***Cq*. *fuscopennata●*** (Gillett 1972, Ochieng et al. 2013), *Ma*. *africana* (Gillett 1972) |
|  | Spondweni virus | *Ae*. *circumluteolus* (Gillett 1972, Jupp 1996), *Ae*. *cumminsi* (Gillett 1972), *Ma*. *africana* (Gillett 1972), *Ma*. *uniformis* (Gillett 1972) |
|  | Tanga virus | *An*. *funestus* (Gillett 1972) |
|  | Uganda S virus | *Ae*. *aegypti* (Gillett 1972) |
|  | Usutu virus | ***Cx*. *pipiens●*** (Ochieng et al. 2013), *Cq*. *aurites* (Haddow et al. 1964, Gillett 1972) |
|  | Wesselsbron virus | *Ae*. *circumluteolus* (Gillett 1972), *Ma*. *uniformis* (Gillett 1972) |
|  | West Nile virus | *Ad*. *africana* (Traore-Lamizana et al. 1994), *Cx*. *neavei* (Traore-Lamizana et al. 1994), ***Cx*. *quinquefasciatus*** (Gillett 1972, Lutomiah et al. 2011), *Cx*. *poicilipes* (Traore-Lamizana et al. 1994), ***Cx*. *univittatus*** (Jupp 1996, Lutomiah et al. 2011, Ochieng et al. 2013), ***Cx*. *vansomereni*** (Lutomiah et al. 2011), *Cq*. *metallicus* (Gillett 1972), *Ma*. *uniformis* (Diallo et al. 2005a), *Mi*. *hispida* (Traore-Lamizana et al. 1994), *Mi*. *splendens* (Traore-Lamizana et al. 1994) |
|  | Yellow fever virus | *Ae*. *aegypti* (Gillett 1972, World Health Organization 2014), *Ae*. *dentatus* (World Health Organization 2014), *Ae*. *furcifer* (World Health Organization 2014), *Ae*. *luteocephalus* (World Health Organization 2014), *Ae*. *metallicus* (Gillett 1972, World Health Organization 2014), *Ae*. *simpsoni* (Gillett 1972, World Health Organization 2014), *Ae*. *tarsalis* (World Health Organization 2014), *Ae*. *vittatus* (Gillett 1972, World Health Organization 2014), *Cx*. *pipiens* (World Health Organization 2014), *Cx*. *thalassius* (World Health Organization 2014), *Cq*. *fuscopennata* (World Health Organization 2014), *Ma*. *africana* (Gillett 1972, World Health Organization 2014) |
|  | Zika virus | *Ae. africanus* (Haddow et al. 1964) |

Names in bold are mosquito species from which pathogen(s) have been isolated in Kenya.

^§^ “Doubtfully or rarely implicated in transmission” (World Health Organization 2013).

● Reported pathogen(s) has been isolated from these mosquitoes in Kenya’s Lake Baringo or Lake Victoria environs.

**References**

**Aranda, C., J. J. Aponte, F. Saute, S. Casimiro, J. Pinto, C. Sousa, V. D. Rosario, V. Petrarca, M. Dgedge, and P. Alonso. 2005.** Entomological characteristics of malaria transmission in Manhica, a rural area in southern Mozambique. J.Med. Entomol. 42: 180–186.

**Chepkorir, E., J. Lutomiah, J.Mutisya, F.Mulwa, K. Limbaso, B. Orindi, and R. Sang. 2014.** Vector competence of *Aedes aegypti* populations from Kilifi and Nairobi for dengue 2 virus and the influence of temperature. Parasite Vector 7: 1–8.

**Crabtree, M., R. Sang, J. Lutomiah, J. Richardson, and B. Miller. 2009.** Arbovirus surveillance of mosquitoes collected at sites of active Rift Valley fever virus transmission: Kenya, 2006-2007. J. Med. Entomol. 46: 961–964.

**Daubney, R. J., and J. R. Hudson. 1933.** Rift Valley fever. E. Afr. Med. J. 10: 2–19.

**Diallo, M., J. Thonnon, M. Traore-Lamizana, and D. Fontenille. 1999.** Vectors of Chikungunya virus in Senegal: Current data and transmission cycles. Am. J. Trop. Med. Hyg. 60: 281–286.

**Diallo, M., P. Nabeth, K. Ba, A. A. Sall, Y. Ba, M. Mondo, L. Girault, M. O. Abdalahi, and C. Mathiot. 2005a.** Mosquito vectors of the 1998-1999 outbreak of Rift Valley fever and other arboviruses (Bagaza, Sanar, Wesselsbron and West Nile) in Mauritania and Senegal. Med. Vet. Entomol. 19: 119-126.

**Diallo, M., A. A. Sall, A. C. Moncayo, Y. Ba, Z. Fernandez, D. Ortiz, L. L. Coffey, C. Mathiot, R. B. Tesh, and S. C. Weaver. 2005b.** Potential role of sylvatic and domestic African mosquito species in dengue emergence. Am. J. Trop.Med. Hyg. 73: 445–449.

**Gillett, J. D. 1972.** Common African mosquitos and their medical importance, William Heinemann, London, United Kingdom.

**Gillies, M. T., and B. De Meillon. 1968.** The Anophelinae of Africa, south of the Sahara. Publ. S. Afr. Inst. Med. Res. 54: 343.

**Gillies, M. T., and M. Coetzee. 1987.** A Supplement to the Anophelinae of Africa South of the Sahara. Publ. S. Afr. Inst. Med. Res. 55: 1–143.

**Gordon, S. W., R. F. Tammariello, K. J. Linthicum, D. J. Dohm, J. P. Digoutte, and M. A. Calvo-Wilson. 1992.** Arbovirus isolations from mosquitoes collected during 1988 in the Senegal River Basin. Am. J. Trop. Med. Hyg. 47: 742–748.

**Haddow, A., M. Williams, J. Woodall, D. Simpson, and L. Goma. 1964.** Twelve isolations of Zika virus from *Aedes* (*Stegomyia*) *africanus* (Theobald) taken in and above a Uganda forest. Bull. World Health Organ. 31: 57.

**Joshi, V., D. T. Mourya, and R. C. Sharma. 2002.** Persistence of dengue-3 virus through transovarial transmission passage in successive generations of *Aedes aegypti* mosquitoes. Am. J. Trop. Med. Hyg. 67: 158–161.

**Jupp, P. 1985.** Culex theileri and Sindbis virus; salivary glands infection in relation to transmission. J. Am. Mosq. Control Assoc. 1: 374.

**Jupp, P. G. 1996.** Mosquitoes of Southern Africa: Culicinae and Toxorhynchitinae, Ekogilde Publishers, Hartebeespoort, South Africa.

**Linthicum, K. J., F. G. Davies, A. Kairo, and C. L. Bailey. 1985.** Rift Valley fever virus (family Bunyaviridae, genus Phlebovirus). Isolations from Diptera collected during an inter-epizootic period in Kenya. J. Hyg.-Cambridge 95: 197–209.

**Logan, T. M., K. J. Linthicum, F. G. Davies, Y. S. Binepal, and C. R. Roberts. 1991.** Isolation of Rift Valley fever virus from mosquitoes (Diptera: Culicidae) collected during an outbreak in domestic animals in Kenya. J. Med. Entomol. 28: 293–295.

**Lutwama, J. J., J. Kayondo, H. M. Savage, T. R. Burkot, and B. R. Miller. 1999.** Epidemic O’Nyong-Nyong fever in southcentral Uganda, 1996–1997: Entomologic studies in Bbaale village, Rakai District. Am. J. Trop. Med. Hyg. 61: 158–162.

**Mwangangi, J. M., E. J. Muturi, S. M. Muriu, J. Nzovu, J. T. Midega, and C. Mbogo. 2013.** The role of *Anopheles arabiensis* and *Anopheles coustani* in indoor and outdoor malaria transmission in Taveta District, Kenya. Parasite Vector 6: 114.

**Njabo, K. Y., A. J. Cornel, C. Bonneaud, E. Toffelmier, R. N. Sehgal, G. Valkiunas, A. F. Russell, and T. B. Smith. 2011.** Nonspecific patterns of vector, host and avian malaria parasite associations in a central African rainforest. Mol. Ecol. 20: 1049–1061.

**Ochieng, C., J. Lutomiah, A. Makio, H. Koka, E. Chepkorir, S. Yalwala, J. Mutisya, L. Musila, S. Khamadi, and J. Richardson. 2013.** Mosquito-borne arbovirus surveillance at selected sites in diverse ecological zones of Kenya; 2007–2012. Virol. J. 10: 140.

**Odhiambo, C., M. Venter, E. Chepkorir, S. Mbaika, J. Lutomiah, R. Swanepoel, and R. Sang. 2014.** Vector competence of selected mosquito species in Kenya for Ngari and Bunyamwera viruses. J. Med. Entomol. 51: 1248–1253.

**Olanga, E. A., L. Okombo, L. W. Irungu, and W. R. Mukabana. 2015.** Parasite vector of malaria on Rusinga Island, Western Kenya. Parasite Vector. 8: 250.

**Omondi, D., D. K. Masiga, Y. U. Ajamma, B. C. Fielding, L. Njoroge, and J. Villinger. 2015.** Unraveling host-vector-arbovirus interactions by two-gene high resolution melting mosquito bloodmeal analysis in a Kenyan wildlife-livestock interface. PLoS ONE 10: e0134375

**Sang, R. C., O. Ahmed, O. Faye, C. L. Kelly, A. A. Yahaya, I. Mmadi, A. Toilibou, K. Sergon, J. Brown, N. Agata, et al. 2008.** Entomologic investigations of a chikungunya virus epidemic in the Union of the Comoros, 2005. Am. J. Trop. Med. Hyg. 78: 77–82.

**Sang, R., E. Kioko, J. Lutomiah, M. Warigia, C. Ochieng, M. O’Guinn, J. S. Lee, H. Koka, M. Godsey, D. Hoel, et al. 2010.** Rift Valley fever virus epidemic in Kenya, 2006/2007: The entomologic investigations. Am. J. Trop. Med. Hyg. 83: 28–37.

**Santa-Ana, M., M. Khadem, and R. Capela. 2006.** Natural infection of *Culex theileri* (Diptera: Culicidae) with *Dirofilaria immitis* (Nematoda: Filarioidea) on Madeira Island, Portugal. J. Med. Entomol. 43: 104–106.

**Seufi, A., and F. Galal. 2010.** Role of *Culex* and *Anopheles* mosquito species as potential vectors of Rift Valley fever virus in Sudan outbreak, 2007. BMC Infect. Dis. 10: 65.

**Tchouassi, D. P., R. Sang, C. L. Sole, A. D. Bastos, L. W. Cohnstaedt, and B. Torto. 2012.** Trapping of Rift Valley fever (RVF) vectors using light emitting diode (LED) CDC traps in two arboviral disease hot spots in Kenya. Parasite Vector 5: 94.

**Traore-Lamizana, M., H. G. Zeller, M. Mondo, J. P. Hervy, F. Adam, and J. P. Digoutte. 1994.** Isolations of West Nile and Bagaza viruses from mosquitoes (Diptera: Culicidae) in central Senegal (Ferlo). J. Med. Entomol. 31: 934–938.

**Turell, M. J., S. M. Presley, A. M. Gad, S. E. Cope, D. J. Dohm, J. C. Morrill, and R. R. Arthur. 1996.** Vector competence of Egyptian mosquitoes for Rift Valley fever virus. Am. J. Trop. Med. Hyg. 54: 136–139.

**Turell, M. J., K. J. Linthicum, L. A. Patrican, F. G. Davies, A. Kairo, and C. L. Bailey. 2008.** Vector competence of selected African mosquito (Diptera: Culicidae) species for Rift Valley fever virus. J. Med. Entomol. 45: 102–108.

**Ughasi, J., H. E. Bekard, M. Coulibaly, D. Adabie-Gomez, J. Gyapong, M. Appawu, M. D. Wilson, and D. A. Boakye. 2012.** *Mansonia africana* and *Mansonia uniformis* are vectors in the transmission of *Wuchereria bancrofti* lymphatic filariasis in Ghana. Parasite Vector 5: 89.

**Wilkes, T. J., Y. G. Matola, and J. D. Charlwood. 1996.** *Anopheles rivulorum*, a vector of human malaria in Africa. Med. Vet. Entomol. 10: 108–110.

**World Health Organization. 2013.** Lymphatic filariasis: a handbook of practical entomology for national lymphatic filariasis elimination programmes, World Health Organization, Geneva.

**World Health Organization. 2014.** Yellow fever: rapid field entomological assessment during yellow fever outbreaks in Africa: Handbook: Methodological field approaches for scientists with a basic background in entomology. World Health Organization, Geneva.
