# Supplementary material for: Composition and Genetic Diversity of Mosquitoes (Diptera: Culicidae) on Islands and Mainland Shores of Kenya’s Lakes Victoria and Baringo
Source: J Med Entomol. 2016 Jul 11;53(6):1348–63. doi: 10.1093/jme/tjw102 (PMC5106823; doi:10.1093/jme/tjw102)
Supplement: Supp. Table 1 [file suppl_data_02.zip › New Supplementary Table 2.docx]

**Supplementary Table 2** The exact location, identity (ID) and GenBank accession numbers of mosquito species on the ITS2 phylogenetic tree

| No. | Morphological ID | Lake site | Exact location | ID on ITS2 tree | Accession no. |
| --- | --- | --- | --- | --- | --- |
| 1 | *Aedeomyia africana* | Lake Baringo | Logumgum | 2A11 | KU056485 |
| 2 | *Aedeomyia africana* | Lake Baringo | Logumgum | 2B01 | KU056486 |
| 3 | *Aedes aegypti* | Lake Victoria | Mfangano Island | 1B06 | KU056487 |
| 4 | *Aedes aegypti* | Lake Victoria | Mfangano Island | 1D08 | KU056488 |
| 5 | *Aedes aegypti* | Lake Victoria | Mbita | M31 | KU056489 |
| 6 | *Aedes aegypti* | Lake Victoria | Mfangano Island | 1B07 | KU056490 |
| 7 | *Aedes aegypti* | Lake Victoria | Mfangano Island | 1D07 | KU056491 |
| 8 | *Aedes aegypti*● | Lake Victoria | Mfangano Island | 1D06 | KU056492 |
| 9 | *Aedes metallicus* | Lake Victoria | Mbita | 1F04 | KU056493 |
| 10 | *Aedes metallicus* | Lake Victoria | Mbita | 1F05 | KU056494 |
| 11 | *Aedes metallicus* | Lake Victoria | Mbita | 1F07 | KU056495 |
| 12 | *Aedes metallicus* | Lake Victoria | Mbita | 1F08 | KU056496 |
| 13 | *Aedes metallicus* | Lake Victoria | Mfangano Island | 1D09 | KU056497 |
| 14 | *Aedes hirsutus* | Lake Victoria | Rusinga Island | 2D03 | KU056498 |
| 15 | *Aedes hirsutus* | Lake Victoria | Chamaunga Island | 2B11 | KU056499 |
| 16 | *Aedes hirsutus* | Lake Victoria | Chamaunga Island | 2C02 | KU056500 |
| 17 | *Aedes hirsutus* | Lake Victoria | Chamaunga Island | 2B12 | KU056501 |
| 18 | *Aedes luteocephalus* | Lake Victoria | Mfangano Island | 1C12 | KU056502 |
| 19 | *Aedes luteocephalus* | Lake Victoria | Mfangano Island | 1D02 | KU056503 |
| 20 | *Culex* (*Lutzia*) *tigripes* | Lake Baringo | Ruko | 2B04 | KU056504 |
| 21 | *Culex* (*Lutzia*) *tigripes* | Lake Victoria | Mbita | 1F03 | KU056505 |
| 22 | *Culex* sp. | Lake Victoria | Mbita | 1A07 | KU056506 |
| 23 | *Culex* sp. | Lake Victoria | Mbita | 1A08 | KU056507 |
| 24 | *Culex pipiens* | Lake Victoria | Rusinga Island | 1C01 | KU056508 |
| 25 | *Culex pipiens* | Lake Victoria | Rusinga Island | 1F01 | KU056509 |
| 26 | *Culex pipiens* | Lake Victoria | Ungoye | 1E.03 | KU056510 |
| 27 | *Culex pipiens* | Lake Victoria | Rusinga Island | 1E.09 | KU056511 |
| 28 | *Culex pipiens* | Lake Victoria | Rusinga Island | 1E.10 | KU056512 |
| 29 | *Mimomyia splendens* | Lake Baringo | Kampi ya Samaki | 2B06 | KU056513 |
| 30 | *Mimomyia splendens* | Lake Baringo | Sirata | 2B07 | KU056514 |
| 31 | *Culex annulioris* | Lake Victoria | Mfangano Island | 1C10 | KU056515 |
| 32 | *Culex annulioris* | Lake Victoria | Mfangano Island | 1C11 | KU056516 |
| 33 | *Culex* sp. | Lake Victoria | Ngodhe Island | 2E.01 | KU056517 |
| 34 | *Culex perexiguus* | Lake Baringo | Kampi ya Samaki | 2A06 | KU056518 |
| 35 | *Culex perexiguus* | Lake Baringo | Kampi ya Samaki | 2A09 | KU056519 |
| 36 | *Mansonia africana* | Lake Baringo | Kokwa Island | M21 | KU056520 |
| 37 | *Mansonia africana* | Lake Baringo | Logumgum | A6B | KU056521 |
| 38 | *Mansonia africana* | Lake Baringo | Salabani | M8 | KU056522 |
| 39 | *Mansonia africana* | Lake Baringo | Ngenyin | A1B | KU056523 |
| 40 | *Mansonia africana* | Lake Baringo | Molo River | M19 | KU056524 |
| 41 | *Mansonia africana*● | Lake Baringo | Logumgum | M53 | KU056525 |
| 42 | *Mansonia africana* | Lake Baringo | Nosuguro | M27 | KU056526 |
| 43 | *Mansonia africana* | Lake Baringo | Logumgum | M10 | KU056527 |
| 44 | *Mansonia africana* | Lake Baringo | Logumgum | M9 | KU056528 |
| 45 | *Mansonia africana*● | Lake Baringo | Kokwa Island | M48 | KU056529 |
| 46 | *Mansonia africana* | Lake Baringo | Logumgum | A10B | KU056530 |
| 47 | *Mansonia africana*● | Lake Baringo | Logumgum | b2 | KU056531 |
| 48 | *Mansonia africana*● | Lake Baringo | Salabani | M40 | KU056532 |
| 49 | *Mansonia africana* | Lake Baringo | Logumgum | A7B | KU056533 |
| 50 | *Mansonia africana*● | Lake Baringo | Logumgum | M41 | KU056534 |
| 51 | *Mansonia africana* | Lake Baringo | Logumgum | 1H04 | KU056535 |
| 52 | *Mansonia africana* | Lake Baringo | Logumgum | A9B | KU056536 |
| 53 | *Mansonia africana* | Lake Baringo | Logumgum | 1H02 | KU056537 |
| 54 | *Mansonia africana*● | Lake Victoria | Mbita | M1 | KU056538 |
| 55 | *Mansonia africana* | Lake Baringo | Kokwa Island | M22 | KU056539 |
| 56 | *Mansonia africana*● | Lake Baringo | Molo River | M45 | KU056540 |
| 57 | *Mansonia africana* | Lake Victoria | Chamaunga Island | M34 | KU056541 |
| 58 | *Mansonia africana* | Lake Victoria | Luanda Nyamasare | A4S | KU056542 |
| 59 | *Mansonia africana* | Lake Victoria | Mbita | A3S | KU056543 |
| 60 | *Mansonia africana* | Lake Victoria | Mfangano Island | A7S | KU056544 |
| 61 | *Mansonia africana*● | Lake Victoria | Mbita | M3 | KU056545 |
| 62 | *Mansonia africana*● | Lake Victoria | Ungoye | A2S | KU056546 |
| 63 | *Mansonia africana*● | Lake Victoria | Ungoye | A5S | KU056547 |
| 64 | *Mansonia africana* | Lake Victoria | Mbita | 1C06 | KU056548 |
| 65 | *Mansonia africana* | Lake Victoria | Mbita | M32 | KU056549 |
| 66 | *Mansonia africana*● | Lake Victoria | Chamaunga Island | A6S | KU056550 |
| 67 | *Mansonia africana*● | Lake Victoria | Chamaunga Island | M35 | KU056551 |
| 68 | *Mansonia africana*● | Lake Victoria | Mfangano Island | A9S | KU056552 |
| 69 | *Mansonia africana*● | Lake Victoria | Mbita | A1S | KU056553 |
| 70 | *Mansonia africana* | Lake Victoria | Mbita | M4 | KU056554 |
| 71 | *Mansonia africana* | Lake Victoria | Mbita | M2 | KU056555 |
| 72 | *Mansonia africana* | Lake Victoria | Mfangano Island | M37 | KU056556 |
| 73 | *Mansonia africana*● | Lake Victoria | Chamaunga Island | A10S | KU056557 |
| 74 | *Mansonia africana* | Lake Victoria | Mfangano Island | M36 | KU056558 |
| 75 | *Mansonia africana* | Lake Baringo | Nosuguro | M26 | KU056559 |
| 76 | *Mansonia africana* | Lake Baringo | Logumgum | A5B | KU056560 |
| 77 | *Mansonia africana* | Lake Baringo | Molo River | M20 | KU056561 |
| 78 | *Mansonia africana*● | Lake Victoria | Chamaunga Island | M33 | KU056562 |
| 79 | *Mansonia africana*● | Lake Baringo | Sirata | M38 | KU056563 |
| 80 | *Mansonia uniformis* | Lake Baringo | Ngenyin | A3B | KU056564 |
| 81 | *Mansonia uniformis*● | Lake Victoria | Chamaunga Island | afs | KU056565 |
| 82 | *Mansonia uniformis* | Lake Victoria | Mbita | U1S | KU056566 |
| 83 | *Mansonia uniformis* | Lake Baringo | Kampi ya Samaki | U1B | KU056567 |
| 84 | *Mansonia uniformis*● | Lake Baringo | Logumgum | M55 | KU056568 |
| 85 | *Mansonia uniformis* | Lake Victoria | Mfangano Island | U7S | KU056569 |
| 86 | *Mansonia uniformis* | Lake Baringo | Kampi ya Samaki | M29 | KU056570 |
| 87 | *Mansonia uniformis* | Lake Baringo | Kampi ya Samaki | M30 | KU056571 |
| 88 | *Mansonia uniformis* | Lake Baringo | Logumgum | M11 | KU056572 |
| 89 | *Mansonia uniformis* | Lake Baringo | Logumgum | U10B | KU056573 |
| 90 | *Mansonia uniformis* | Lake Baringo | Logumgum | U7B | KU056574 |
| 91 | *Mansonia uniformis* | Lake Baringo | Molo River | M18 | KU056575 |
| 92 | *Mansonia uniformis* | Lake Baringo | Nosuguro | M24 | KU056576 |
| 93 | *Mansonia uniformis*● | Lake Baringo | Nosuguro | M50 | KU056577 |
| 94 | *Mansonia uniformis* | Lake Baringo | Sirata | M14 | KU056578 |
| 95 | *Mansonia uniformis* | Lake Victoria | Mfangano Island | U8S | KU056579 |
| 96 | *Mansonia uniformis* | Lake Baringo | Logumgum | M12 | KU056580 |
| 97 | *Mansonia uniformis* | Lake Baringo | Logumgum | 1F10 | KU056581 |
| 98 | *Mansonia uniformis* | Lake Baringo | Logumgum | 1F12 | KU056582 |
| 99 | *Mansonia uniformis* | Lake Baringo | Logumgum | 1G02 | KU056583 |
| 100 | *Mansonia uniformis* | Lake Baringo | Logumgum | 1G03 | KU056584 |
| 101 | *Mansonia uniformis*● | Lake Baringo | Logumgum | u2 | KU056585 |
| 102 | *Mansonia uniformis* | Lake Baringo | Logumgum | 1G01 | KU056586 |
| 103 | *Mansonia uniformis* | Lake Victoria | Luanda Nyamasare | 1A09 | KU056587 |
| 104 | *Mansonia uniformis* | Lake Baringo | Logumgum | U6B | KU056588 |
| 105 | *Mansonia uniformis*● | Lake Baringo | Logumgum | unb | KU056589 |
| 106 | *Mansonia uniformis* | Lake Baringo | Kampi ya Samaki | 1F09 | KU056590 |
| 107 | *Mansonia uniformis* | Lake Baringo | Sirata | M16 | KU056591 |
| 108 | *Mansonia uniformis* | Lake Victoria | Chamaunga Island | U6S | KU056592 |
| 109 | *Mansonia uniformis* | Lake Baringo | Kampi ya Samaki | M28 | KU056593 |
| 110 | *Mansonia uniformis*● | Lake Baringo | Logumgum | M42 | KU056594 |
| 111 | *Mansonia uniformis*● | Lake Baringo | Logumgum | U3B | KU056595 |
| 112 | *Mansonia uniformis* | Lake Victoria | Chamaunga Island | uns | KU056596 |
| 113 | *Mansonia uniformis* | Lake Baringo | Logumgum | 1G06 | KU056597 |
| 114 | *Mansonia uniformis* | Lake Baringo | Logumgum | U2B | KU056598 |
| 115 | *Mansonia uniformis* | Lake Baringo | Logumgum | U9B | KU056599 |
| 116 | *Mansonia uniformis* | Lake Baringo | Logumgum | U8B | KU056600 |
| 117 | *Mansonia uniformis* | Lake Baringo | Nosuguro | M23 | KU056601 |
| 118 | *Mansonia uniformis*● | Lake Baringo | Logumgum | M43 | KU056602 |
| 119 | *Mansonia uniformis* | Lake Baringo | Molo River | M17 | KU056603 |
| 120 | *Mansonia uniformis* | Lake Baringo | Logumgum | M13 | KU056604 |
| 121 | *Mansonia uniformis* | Lake Baringo | Nosuguro | M25 | KU056605 |
| 122 | *Mansonia uniformis* | Lake Baringo | Logumgum | U5B | KU056606 |
| 123 | *Mansonia uniformis* | Lake Baringo | Logumgum | 1F11 | KU056607 |
| 124 | *Mansonia uniformis* | Lake Baringo | Logumgum | 1G04 | KU056608 |
| 125 | *Mansonia uniformis* | Lake Baringo | Sirata | M15 | KU056609 |
| 126 | *Culex duttoni* | Lake Baringo | Ruko | 1H11 | KU056610 |
| 127 | *Anopheles gambiae* | Lake Baringo | Kampi ya Samaki | 2A03 | KU056611 |
| 128 | *Anopheles gambiae* | Lake Baringo | Sirata | 2A01 | KU056612 |
| 129 | *Anopheles gambiae* | Lake Victoria | Rusinga Island | 1A01 | KU056613 |
| 130 | *Anopheles gambiae* | Lake Victoria | Rusinga Island | 1A03 | KU056614 |
| 131 | *Anopheles gambiae* | Lake Victoria | Rusinga Island | 1A04 | KU056615 |
| 132 | *Anopheles gambiae* | Lake Victoria | Mbita | 1D11 | KU056616 |
| 133 | *Anopheles gambiae* | Lake Baringo | Sirata | 2A02 | KU056617 |

●Entries that do not have corresponding COI gene sequences
